# Current rat models of extracorporeal life support following global ischaemia: a scoping review

**DOI:** 10.1186/s40635-026-00869-6

**Published:** 2026-02-19

**Authors:** Johannes Dinkelaker, Jan-Steffen Pooth, Georg Trummer, Sonja Bröer, Hans-Jörg Busch, Marius Schimmel, Jakob Wollborn, Sam Joé Brixius

**Affiliations:** 1 Center for Experimental Models and Transgenic Service CEMT-FR, Medical Center, Faculty of Medicine, University of Freiburg, Breisacher Str. 66, 79106 Freiburg, Germany; 2 Department of Emergency Medicine, Medical Center, Faculty of Medicine, University of Freiburg, Hugstetter Strasse 55, 79106 Freiburg, Germany; 3 Institute of Pharmacology and Toxicology, School of Veterinary Medicine, Freie Universität Berlin, Berlin, Germany; 4 Department of Anesthesiology, Mass General Brigham, Brigham and Women’s Hospital, Harvard Medical School, Boston, USA; 5 Department of Cardiovascular Surgery, Faculty of Medicine, University Heart Center Freiburg-Bad Krozingen, University of Freiburg, Hugstetter Strasse 55, 79106 Freiburg, Germany

**Keywords:** Rats, Global ischaemia, Cardiac arrest, Extracorporeal life support, Extracorporeal resuscitation, Reperfusion, Cardiopulmonary resuscitation, Ventricular fibrillation, Acute care medicine

## Abstract

**Introduction:**

Small animal models are indispensable in cardiovascular research. This scoping review aims to provide an overview of contemporary rat models for extracorporeal life support (ECLS) after global ischaemia.

**Material and methods:**

A systematic search was conducted in PubMed, Web of Science and Embase to identify studies involving rat models of global ischaemia followed by ECLS from January 2000 to December 2024. Title and abstracts were screened by two independent reviewers, and the remaining full text was included in predefined data extraction forms.

**Results:**

A total of 79 studies met the inclusion criteria. Male Sprague Dawley rats were predominantly used (82%), with limited reporting on animal age and inconsistent use of analgesia. The majority of studies employed ECLS configurations with roller pumps (71%), custom-made oxygenators (41%), venous drainage via the jugular vein (96%) and arterial inflow via the femoral (53%) or caudal (35%) artery. Three distinct clinical scenarios were identified: extracorporeal cardiopulmonary resuscitation (41%), emergency preservation and resuscitation (13%), and deep hypothermic circulatory arrest (47%). Substantial methodological heterogeneity was observed across models, particularly in ischaemia induction, ECLS protocols, outcome measures, and reporting standards.

**Conclusion:**

Rat models are increasingly used in ECLS research and offer valuable opportunities for investigating pathophysiological mechanisms and advantages for translational studies. To utilize their full potential, improved standardization and adherence to existing guidelines are needed to enhance their reproducibility and clinical relevance.

**Supplementary Information:**

The online version contains supplementary material available at 10.1186/s40635-026-00869-6.

## Introduction

Extracorporeal life support (ECLS) has become an increasingly important option across various clinical scenarios involving severe circulatory and respiratory failure or cardiogenic shock, including but not limited to cardiopulmonary bypass (CPB), extracorporeal cardiopulmonary resuscitation (ECPR) and trauma resuscitation [13, 24].

Among these, ECPR has gained particular attention as a promising intervention for patients experiencing cardiac arrest (CA) refractory to conventional cardiopulmonary resuscitation [1, 32]. The growing clinical relevance of ECPR is reflected in its expanding use and the ongoing efforts to optimize patient selection, timing, and implementation strategies [3, 32]. To investigate the underlying mechanisms and therapeutic potential, animal models have become essential tools in preclinical research [5]. Systematic reviews to summarize and compare these models have been conducted, showing a predominant use of large animal models [14, 16]. While these have traditionally dominated the field, the emergence of rat models offers a complementary approach that may help address specific translational questions [17].

Moreover, ECLS models have been increasingly employed in other experimental domains, particularly in trauma resuscitation and cardiopulmonary bypass (CPB). In the CPB setting, they enable controlled analysis of the haemodynamic, inflammatory, and coagulative alterations associated with extracorporeal circulation during cardiac surgery. This facilitates mechanistic insights into the complex interplay between systemic inflammation, microcirculatory disturbances, and end-organ dysfunction, thereby supporting the development and refinement of novel perfusion strategies and circuit technologies [18].

In addition, ECLS has been integrated as a component of comprehensive trauma resuscitation strategies, where it provides cardiopulmonary support in the setting of severe haemorrhagic shock, thoracic injury, or trauma-associated cardiac arrest when conventional measures are insufficient. Experimental trauma research has traditionally relied on large-animal models, but well-characterized small-animal platforms have also been developed to reproduce key elements of trauma-associated coagulopathy and organ injury without extracorporeal support, such as rat polytrauma and haemorrhage models [7]. Building on these foundations, experimental ECMO models of traumatic haemorrhagic shock have demonstrated that extracorporeal circulation can stabilize haemodynamics, improve oxygen delivery, and partially correct acidosis and coagulopathy during early resuscitation [20]. In parallel, clinical data syntheses indicate that extracorporeal life support is increasingly used as an adjunct for gas exchange and circulatory support in selected trauma patients with otherwise refractory shock or respiratory failure [21]. Together, these experimental and clinical ECLS models do not aim to replicate the typical course of shock in all severely injured patients, but rather provide a standardized framework to dissect how extracorporeal flow, oxygenation, and circuit-induced inflammation modify the cardiovascular and microcirculatory response to severe shock and reperfusion. By enabling controlled comparisons between supported and non-supported resuscitation, they help to separate ECLS-specific effects from the underlying injury response and thereby support the evaluation of targeted resuscitative strategies aimed at restoring organ perfusion and mitigating ischaemia–reperfusion injury.

Several rat models employing extracorporeal life support following global ischaemia have been published, aiming to investigate the pathophysiological processes observed in human ECLS scenarios. However, despite their growing use, the scope, methodological diversity, and translational relevance of these models have not yet been systematically reviewed.

Given the potential of these models to inform clinical practice and to contribute to the principles of the 3Rs (Replacement, Reduction, Refinement of animal models) by enabling targeted and ethically optimized research, a comprehensive overview of existing approaches is needed. This is particularly important considering their versatile applications and the increasing need for robust preclinical data to guide clinical translation.

The current scoping review aims to identify, summarize and analyse rat models of extracorporeal life support after global ischaemia, with particular focus on their methodology, endpoints and animal welfare. It is intended to guide the selection of appropriate animal models in order to improve the clinical translatability of preclinical research for extracorporeal life support while reducing the number of large animals required.

## Results

### Included articles: origin and time trends

**Fig. 1:**
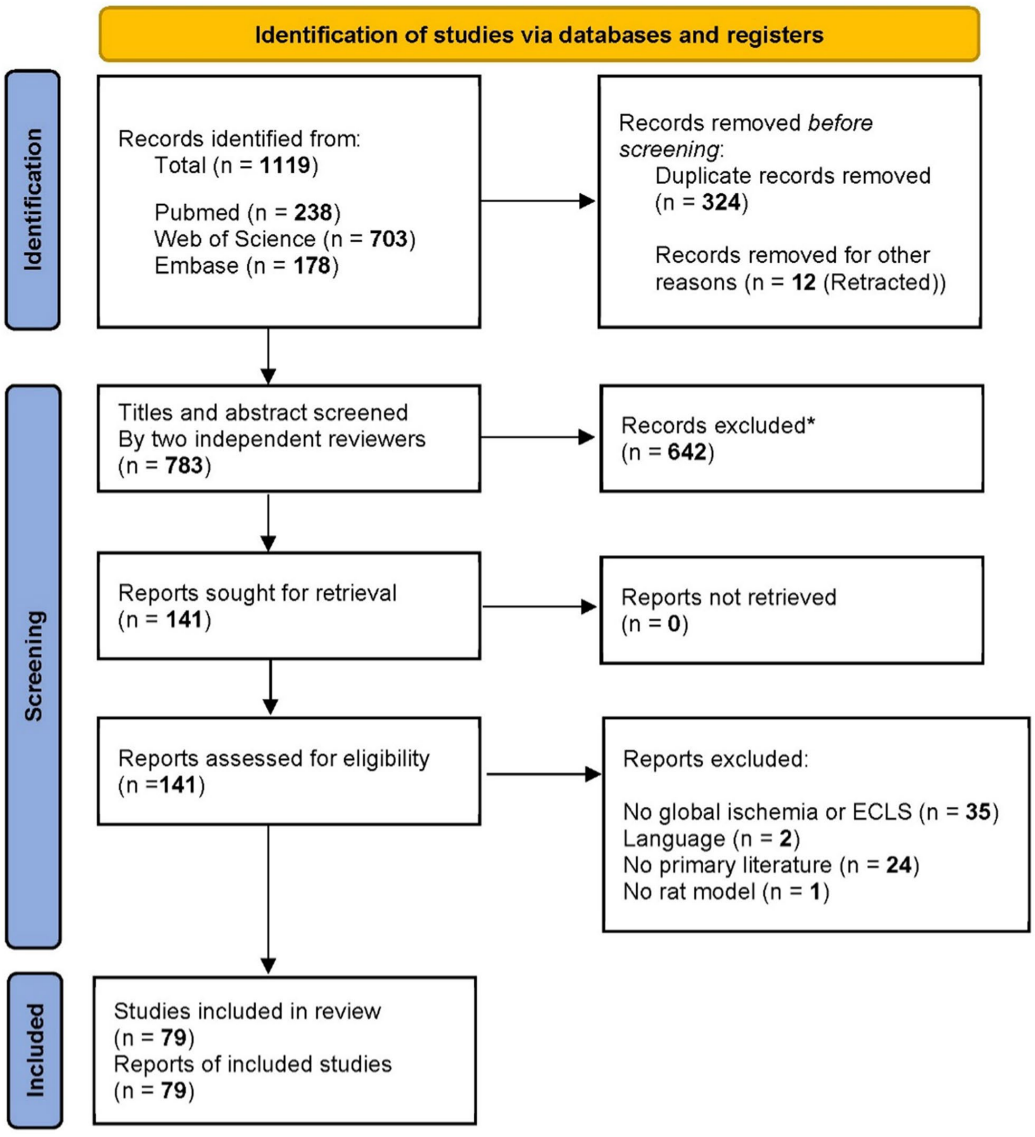
PRISMA flow diagram for study selection. Of 1119 records identified, 79 studies were included after screening. PRISMA, Preferred Reporting Items for Systematic Reviews and Meta-Analyses; ECLS, Extracorporeal Life Support. *No automation tools were used; all records were excluded by a human

The literature search yielded a result of 1119 articles (PubMed: 238; Web of Science: 703; Embase: 178, see Fig. 1). After removing 324 duplicate records and 12 retracted publications, 783 records remained for title and abstract screening. Of these, 642 records did not meet the predefined inclusion criteria and were excluded. The inter-rater agreement between the two reviewers was high, with a Cohen’s kappa coefficient of 0.90.

Subsequently, 141 full-text articles were retrieved and assessed for eligibility (see Fig. 1).

Of these, 62 studies were excluded for the following reasons: absence of global ischaemia or ECLS (*n* = 35), non-English language (*n* = 2), non-primary literature (*n* = 24), or use of a non-rat model (*n* = 1). Ultimately, 79 studies met the inclusion criteria and were included in the review.

**Fig. 2:**
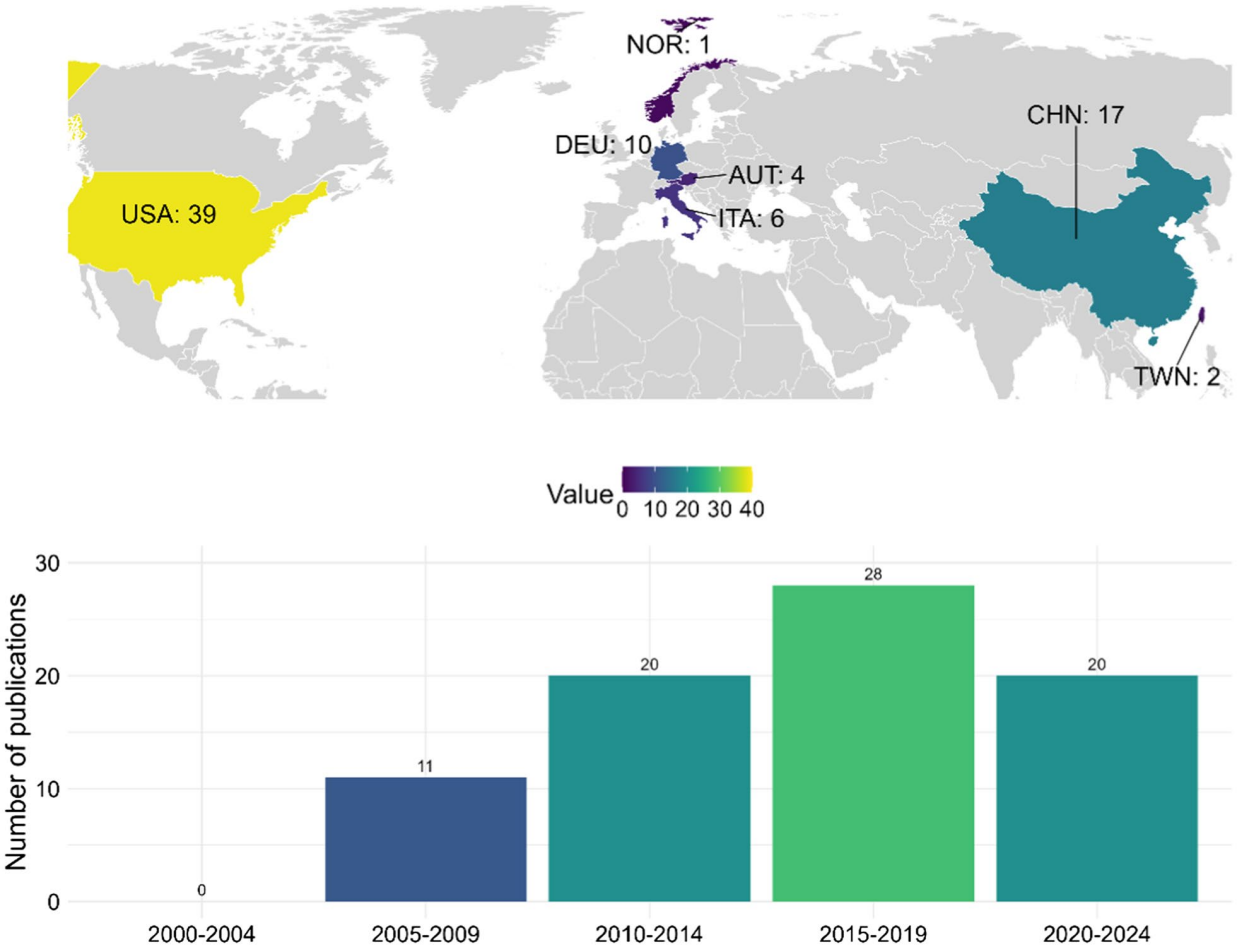
Publications per year and country of origin. The top panel shows a world map highlighting the number of publications per country. The colour scale represents the number of publications. The bottom panel is showing the number of publications across 5-year periods from 2000 to 2024. USA, United States of America; CHN, China; NOR, Norway; DEU, Germany; ITA, Italy; AUT, Austria; TWN, Taiwan

The number of publications in this field shows an upward trend, from 2000–2004, when no publications were recorded, to a peak in 2015–2019 with 29 publications (see Fig. 2). In the most recent period (2020–2024), the number of publications slightly declined to 20.

Geographically, the 79 included studies originated from seven countries across North America, Europe and Asia. The United States contributed the largest share with 39 publications, followed by China (*n* = 17) and Germany (*n* = 10). In total, European countries accounted for 21 publications.

### Animal characteristics and group composition

Table 1 summarizes the characteristics of the animals used and the composition of the experimental groups. The vast majority of studies (96%) used male animals, predominantly Sprague Dawley rats (82%), with only a single study including both sexes. Reporting of age and weight was inconsistent, with 72% of studies not specifying animal age, while weight was more frequently reported (95%), showing a median minimum of 400 g and a maximum of 450 g. Most animals were healthy wild-type animals (96%), while only 3% were genetically modified. The number of groups per study and group sizes varied considerably, with a median of three groups (interquartile range (IQR): 2–4), and a median of 8 animals (IQR: 6–10) per group in the smallest intervention group. A SHAM group—serving as a procedural control without the primary intervention—was included in 51 studies (65%). Randomization, defined as the allocation of animals to groups using a random method, was reported in 53 studies (67%), whereas blinding was almost entirely absent, reported in only one study (1%).

**Table 1:** Animal characteristics and group composition

Variable	All studies (*n* = 79)
**Animal**	
Number of animals—median (quartiles)	28 (18, 34)
**Species—n (%)**	
Sprague Dawley	65 (82)
Wistar	12 (15)
Other or not specified	2 (3)
**Sex—n (%)**	
Male	76 (96)
Female	0 (0)
Both	1 (1)
Not specified	2 (3)
**Age [weeks]—n (%)**	
< 10	5 (6)
10–12	5 (6)
12–14	8 (10)
14–16	2 (3)
16–18	2 (3)
> 18	0 (0)
Not specified	57 (72)
**Weight [g]—median (quartiles)**	
Min	400 (350, 445)
Max	450 (400, 500)
**Morbidity—n (%)**	
Normal animals	76 (96)
Genetically modified	2 (3)
Not specified	1 (1)
**Groups**	
Number of groups—median (quartiles)	3 (2, 4)
Number of animals in smallest intervention group—median (quartiles)	8 (6, 10)
Animals with global ischaemia and ECLS—median (quartiles)	18 (10, 30)
**SHAM Group—n (%)**	
No/not reported	28 (35)
Yes	51 (65)
Randomization*—n (%)	
No/not reported	21 (28)
Yes	53 (72)
Blinding*—n (%)	
No/not reported	78 (99)
Yes	1 (1)

*n* number, *Min* minimum, *Max* maximum

^*^not applicable in 5 studies because only one group was used

### Baseline characteristics

Among the 79 studies analysed, several key methodological features were identified. The baseline characteristics are provided in Table 2.

**Table 2:** Baseline characteristics

Variable	All studies (*n* = 79)
**Animals fasted—n (%)**	
No/not reported	66 (84)
Yes	13 (16)
** Anaesthesia—n (%)**	79 (100)
Inhalation	52 (66)
Inhalation and intraperitoneal	11 (14)
Inhalation and intravenous	8 (10)
Intraperitoneal	4 (5)
Intravenous and intraperitoneal	1 (1)
Not reported	3 (4)
**Pain medication—n (%)**	
No/not reported	48 (61)
Yes	31 (39)
Fentanyl	18 (58)
Buprenorphine	6 (19)
Ketoprofen	2 (6)
Ketamine	1 (3)
Fentanyl and ketamine	1 (3)
Piritramide	1 (3)
Piritramide and carprofen	1 (3)
Not reported	1 (3)
**Muscle relaxation—n (%)**	
No/not reported	49 (62)
Yes	30 (38)
Relaxation without reported pain medication	19 (63)
**Monitoring—n (%)**	
Arterial blood pressure	76 (96)
Temperature	73 (92)
Blood gas analysis	72 (91)
Electrocardiography	53 (67)
Central venous pressure	20 (25)
Pulse oximetry	10 (13)
Cardiac output	5 (6)
Electroencephalography	1 (1)
Somatosensory evoked potentials	0 (0)
**Baseline measurements before ischaemia—n (%)**	
Yes	43 (54)
No/not reported	36 (46)

*n* number

Inhalation anaesthesia was the predominant method, used in 52 studies (66%), while combinations with intraperitoneal or intravenous routes were reported less frequently (14%). All studies used some form of anaesthesia, with only 31 (39%) explicitly reporting the use of analgesics. The analgesic agents used included fentanyl (58%), buprenorphine (19%) or piritramide (3%), as well as non-steroidal anti-inflammatory drugs (NSAID) like ketoprofen and carprofen or ketamine as N-methyl-D-aspartate (NMDA) receptor antagonist. In addition, combined use of analgesics was documented in two single cases for piritramide with carprofen and fentanyl with ketamine. Muscle relaxation was reported in 30 studies (38%), and notably, in nearly two-thirds of those cases (63%), no concurrent pain medication was described.

Monitoring of basic physiological parameters was common, with arterial blood pressure (96%), temperature (92%), and blood gas analysis (91%) being the most frequently recorded. Electrocardiography was reported in 53 studies (67%), while more advanced monitoring methods such as central venous pressure (25%), pulse oximetry (13%), and cardiac output (6%) were less frequently employed. Notably, functional neurological monitoring was virtually absent, with only one study reporting the use of electroencephalography and none employing somatosensory evoked potentials or regional cerebral blood flow monitoring.

### Technical ECLS setup

The design of the ECLS system used was heterogeneous across the studies (see Table 3). The typical configuration consisted of a roller or peristaltic pump (98%), a venous reservoir (89%), venous drainage via the jugular vein (96%), and arterial return through either the femoral artery (53%), the caudal artery (35%), or the carotid artery (11%).

**Table 3:** Model characteristics

Variable	All studies (*n* = 79)
**Modelled clinical scenario—n (%)**	
Extracorporeal cardiopulmonary resuscitation (ECPR)	32 (41)
Emergency preservation and resuscitation (EPR)	10 (13)
Deep hypothermic circulatory arrest (DHCA)	37 (47)
**Technical specifications of extracorporeal life support (ECLS)**	
** Cannulation sites for ECLS—n (%)**	
Art	
Art. Femoralis	42 (53)
Art. Carotis communis	9 (11)
Tail or caudal artery	28 (35)
Ven	
Ven. Jugularis	76 (96)
Right atrium	3 (4)
** Venous reservoir—n (%)**	
Yes	70 (89)
No/not reported	9 (11)
** Type of pump used—n (%)**	
Roller pump	56 (71)
Peristaltic pump	21 (27)
Other/not specified	2 (3)
** Used oxygenator—n (%)**	
Custom made	32 (41)
Commercially available	25 (32)
Not specified	22 (28)
** Extracorporeal flow measured—n (%)**	
Yes	7 (9)
No/not reported	72 (91)

*n* number, *Art.* arterial, *Ven.* venous, *ECPR* extracorporeal cardiopulmonary resuscitation, *EPR* emergency preservation and resuscitation, *DHCA* deep hypothermic circulatory arrest, *ECLS* extracorporeal life support

### Clinical scenario

Three different clinical scenarios were modelled across the studies: extracorporeal cardiopulmonary resuscitation (ECPR) in 32 studies (41%), emergency preservation and resuscitation (EPR) in 10 studies (13%), and deep hypothermic circulatory arrest (DHCA) in 37 studies (47%).

Table 4 summarizes the methods used for ischaemia induction across the different experimental models. A comprehensive comparison across all included studies is provided in Supplement 8. Global ischaemia was induced using hypothermia in 35 studies (44%), asphyxia in 22 studies (28%), electrical induction in 9 studies (11%), and cardioplegia in 13 studies (16%). In all EPR models, haemorrhage was performed prior to cardiac arrest induction, most commonly followed by cardioplegia (90%). In DHCA models, hypothermia was predominantly used to induce circulatory arrest (95%). In ECPR models, cardiac arrest was most frequently induced by asphyxia (69%), followed by electrical induction (25%). Notably, in 43 (54%) of all studies, no explicit definition of ischaemia was provided.

**Table 4:** Comparative characteristics of experimental procedures

Variable	ECPR (*n* = 32)	EPR* (*n* = 10)	DHCA (*n* = 37)
**Induction and ischaemia**			
FiO2 before CA—% (quartiles)	50 (30, 93)	25 (25, 25)	50 (45, 100)
**Technique of induction of global ischaemia—n (%)**			
Hypothermia	0 (0)	0 (0)	35 (95)
Asphyxia	22 (69)	0 (0)	0 (0)
Electric	8 (25)	1 (10)	0 (0)
Cardioplegia	2 (6)	9 (90)	2 (5)
**Clear definition of ischaemia—n (%)**			
Yes	20 (63)	1 (10)	15 (41)
No	12 (38)	9 (90)	22 (59)
**Maximum no-flow time [min]**			
Median (quartiles)	12 (8, 20)	20 (20, 75)	45 (41, 60)
Minimum—maximum	4–30	1–80	1–105
**Extracorporeal circulation**			
** Maximum Duration of ECLS [min]**			
Median (quartiles)	30 (30, 60)	60 (60, 60)	60 (30, 90)
Minimum—maximum	10–180	60–80	30–120
** Type of priming—n (%)**			
Balanced electrolyte solution	17 (53)	0 (0)	0 (0)
Colloid solution	3 (9)	0 (0)	23 (62)
Combined	7 (22)	1 (10)	6 (16)
Different groups	1 (3)	0 (0)	1 (3)
Blood	0 (0)	9 (90)	0 (0)
Not reported	4 (13)	0 (0)	7 (19)
Priming volume [ml]—median (quartiles)	14 (12, 20)	13 (13, 13)	10 (10, 13)
** Blood transfusion—n (%)**			
Yes	6 (19)	10 (100)	3 (8)
No/not reported	26 (81)	0 (0)	34 (92)
** Active cooling—n (%)**			
Yes	4 (13)	9 (90)	37 (100)
Flush after cardiac arrest	0 (0)	9 (100)	0 (0)
Continuous cooling	4 (100)	0 (0)	37 (100)
No/not reported	28 (88)	1 (10)	0 (0)
** Maximum extracorporeal target flow [ml/kg/min]—n (%)**			
< 50	2 (6)	0 (0)	0 (0)
50–90	5 (16)	0 (0)	1 (3)
90–130	9 (28)	1 (10)	9 (24)
130–170	10 (31)	0 (0)	3 (8)
170–190	0 (0)	4 (40)	15 (41)
Not reported	6 (19)	5 (50)	9 (24)
** Drugs used during ECLS—n (%)**			
Sodium bicarbonate	15 (47)	3 (30)	13 (35)
Norepinephrine	5 (16)	0 (0)	8 (22)
Trometamol	4 (13)	0 (0)	5 (14)
Epinephrine	8 (25)	0 (0)	0 (0)
Other**	4 (13)	3 (30)	4 (11)
**Endpoint—n (%)**			
Survival	10 (31)	9 (90)	22 (59)
Neurological outcome	16 (50)	10 (100)	24 (65)
Cardiological outcome	16 (50)	3 (30)	9 (24)
Renal outcome	2 (6)	0 (0)	2 (5)
Other	12 (38)	0 (0)	6 (16)

*ECPR* extracorporeal cardiopulmonary resuscitation, *EPR* emergency preservation and resuscitation, *DHCA* deep hypothermic circulatory arrest, *n* number; min, minutes, *ml* millilitres, *ECLS* extracorporeal life support

^*^All models performed haemorrhage before induction of ischaemia

**see supplement for details;

One of the most prominent differences among the models was observed in the maximum no-flow time, defined as the interval of complete circulatory arrest without perfusion, which varied significantly depending on the clinical scenario. ECPR models demonstrated the shortest no-flow intervals, with a median of 12 min (IQR 8–20; range 4–30 min). In contrast, EPR models exhibited substantially longer and more variable no-flow times (median 20 min, IQR 20–75; maximum 80 min). DHCA models also involved prolonged no-flow periods, with a median duration of 45 min (range 1–105 min).

Priming volumes for the extracorporeal circuit varied according to experimental design and study objectives, with median volumes ranging between 10 and 14 mL. Priming solutions included balanced electrolyte solutions, colloids, or blood products—with blood-based priming used in nearly all EPR models.

Active cooling was employed in nearly all DHCA and EPR studies, including both hypothermia-based ischaemia models and protocol-driven temperature management strategies. In contrast, active cooling was not implemented in 28 ECPR studies (88%).

Target extracorporeal flow rates varied considerably: 20 studies (25%) did not specify a target flow and only 7 studies (9%) directly measured extracorporeal blood flow, as shown in Table 3.

The overall median duration of ECLS support across all studies was 60 min (IQR 30–60), with marked variability between model types. ECPR models had a shorter median support time of 30 min (range 10–180 min). DHCA models reported ECLS durations between 30 and 120 min, while EPR models were more consistent, ranging from 60 to 80 min, with a shared median of 60 min.

Blood transfusion on ECLS support was reported in 19 studies (24%), predominantly in EPR-related models (100%, all 10 studies), likely facilitated by the availability of autologous blood from the induction procedure. In contrast, transfusions were used far less frequently in ECPR (19%) and nearly absent in DHCA (8%) models. Pharmacological management on ECLS varied considerably. While buffering agents such as sodium bicarbonate and vasoactive agents like norepinephrine were commonly administered, many studies reported either a limited use of pharmacologic interventions or did not report drug administration at all.

Endpoints differed notably between model types. Survival endpoints were most frequently assessed in EPR models (90%) and DHCA models (59%), while being less commonly reported in ECPR models (31%). In this review, survival was defined as any awakening from anaesthesia. This definition may differ from those used in the original studies. Animals that remained under anaesthesia were not considered survivors.

Neurological outcome was the most frequently reported endpoint, assessed in 63% of studies, followed by cardiac (35%) and renal outcomes (5%). Neurological assessments included a variety of methods: clinical scoring systems (e.g., neurological deficit scores), histological or biochemical analyses, imaging techniques, electrophysiological evaluations and in some cases, combinations. Neurological assessments were particularly prevalent in EPR models (100%) and DHCA models (65%).

In contrast, cardiac outcomes were less frequently assessed overall but were reported at the same rate as neurological outcomes in ECPR models (50% each). Cardiac evaluation methods included haemodynamic monitoring, blood gas analysis, and histological or biochemical assessments. Some studies also employed imaging or electrophysiological measures.

Renal outcomes were rarely assessed and were reported in only 4 out of 79 studies (5%).

### Model-specific methodological patterns

Given the substantial heterogeneity across rat ECLS models, we performed a model-specific comparative synthesis stratified by the primary experimental objective and translational intent of each study (ECPR, EPR, DHCA). This approach enables within-scenario comparisons and avoids conflating fundamentally different pathophysiological paradigms.

ECPR involved whole-body ischaemia followed by the initiation of extracorporeal life support, aiming to provide complete circulatory support (see Table 5). Notably, none of the included studies implemented basic or advanced life support measures prior to the initiation of extracorporeal life support, indicating that ECLS was uniformly used as the primary resuscitative method. Defibrillation was reported in only five studies (16%) and was exclusively applied in models where electrical induction of cardiac arrest was used, indicating differences in underlying cardiac rhythms during reperfusion depending on the induction method. Return of spontaneous circulation (ROSC) was defined as an explicit experimental goal in less than half of the studies (41%), and among these, only five studies (38%) provided a clear and reproducible definition of ROSC.

**Table 5:** Characteristics of ECPR models

Variable	ECPR (*n* = 32)
**Basic or advanced life support prior to ECLS—n (%)**	
Yes	0 (0)
No	32 (100)
**Defibrillation—n (%)**	
No/not reported	27 (84)
Yes	5 (16)
**ROSC as experimental goal—n (%)**	13 (41)
Clear definition of ROSC	5 (38)

*ECPR* extracorporeal cardiopulmonary resuscitation, *ECLS* extracorporeal life support, *n* number, *ROSC* return of spontaneous circulation

DHCA modelled surgical arrest using deep hypothermia for organ protection during prolonged ischaemia or accidental hypothermia. If more than one method of ischaemia induction was used, the final and predominant technique was recorded.

Deep hypothermic circulatory arrest (DHCA) models demonstrated a high degree of methodological consistency with respect to temperature management (see Table 6). The vast majority of studies targeted profound hypothermia, with 84% aiming for core temperatures between 15 °C and 19 °C, reflecting established surgical DHCA protocols. Only a small minority employed temperatures below 15 °C or above 24 °C, while two studies investigated different temperature groups. Rewarming was generally standardized, with a median target temperature of 35.5 °C, and rewarming durations exceeding cooling times (median 40 vs. 30 min).

**Table 6:** Characteristics of DHCA models

Variable	DHCA (*n* = 37)
**Target DHCA temperature [C°]—n (%)**	
< 15	3 (8)
15–19	31 (84)
> 24	1 (3)
Different groups	2 (5)
**Target rewarming temperature [C°]—median (quartiles)**	35.5 (34, 36)
**Place of temperature measurement—n (%)**	
Rectal	19 (51)
Pericranial	7 (19)
Rectal and pericranial	7 (19)
Oesophageal and nasopharyngeal	2 (5)
Other* or not reported	2 (5)
**Continuous cooling technique—n (%)**	
Topical cooling	6 (16)
Extracorporeal heat exchanger	12 (32)
Combined	8 (22)
Not reported	11 (30)
**Time [min]—median (quartiles)**	
Cooling time	30 (30, 30)
Rewarming time	40 (30, 60)
Cardioplegia—n (%)	2 (5)

*DHCA* deep hypothermic circulatory arrest, *n* number, *min* minutes, *CA* cardiac arrest, *ROSC* return of spontaneous circulation, *ECLS* extracorporeal life support

^*^Vena jugularis and rectal

Temperature monitoring sites varied considerably, with rectal measurements being most common (51%), followed by pericranial or combined rectal–pericranial approaches, underscoring heterogeneity in surrogate markers for cerebral temperature. Continuous cooling techniques were inconsistently reported; when specified, heat exchangers were most frequently used, either alone or in combination with topical cooling. Furthermore, actual rewarming flow rates were not explicitly reported in DHCA models; instead, studies either described a gradual increase in extracorporeal flow during the rewarming phase or referred only to the general ECLS target flow. This lack of detailed flow reporting limits the comparability of rewarming strategies across studies. Cardioplegia was rarely employed, reported in only two studies (5%), further indicating that most DHCA models relied primarily on hypothermia alone for the induction of CA.

EPR modelled trauma-related CA with preceding controlled haemorrhage and rapid ECLS deployment as a salvage intervention. All EPR models included haemorrhage prior to CA induction; in nine cases, this was combined with cardioplegia, and in one case, with electrical induction of CA.

Emergency preservation and resuscitation (EPR) models were characterized by a highly standardized haemorrhage–flush–reperfusion sequence designed to simulate traumatic cardiac arrest under extreme ischaemic conditions (Table 7). Blood loss was quantified in nearly all studies, predominantly as an absolute volume (90%), with a median maximum blood loss of 12.5 mL achieved within a short and uniform time frame of 5 min. No model incorporated additional traumatic injury beyond controlled haemorrhage, underscoring the primary focus on ischaemia–reperfusion physiology rather than polytrauma.

**Table 7:** Comparative characteristics of EPR models

Variable	EPR (*n* = 10)
**Blood loss**	
** Quantified—n (%)**	
Absolute	9 (90)
Relative	1 (10)
** Maximum volume [ml]—median (quartiles)**	12.5 (12.5, 12.5)
** Blood loss time [min]—median (quartiles)**	5 (5, 5)
** Additional trauma—n (%)**	
Yes	0 (0)
No/not reported	10 (100)
**Flush-cooling-protocol**	
Flush volume [ml]—median (quartiles)	270 (270, 270)
Flow [ml/min]—median (quartiles)	50 (50, 50)
** Temperature—n (%)**	
Ice cold	4 (40)
Room temperature	1 (10)
Different groups	4 (40)
Not reported	1 (10)
** Composition—n (%)**	
Crystalloid	9 (90)
Mixed*	1 (10)
** Temperature target [C°]—median (quartiles)**	
CA and flush	15 (15, 15)
ECLS	34 (34,0, 34,5)
**Time [min]—median (minimum—maximum)**	
Haemorrhagic shock after blood loss	0 (0–35)
CA until flush-cooling	1 (0–6)
Flush until ECLS	32,5 (14–75)

*EPR* emergency preservation and resuscitation, *n* number, *min* minutes, *ml* millilitres, *°C* degrees Celsius, *ECLS* extracorporeal life support, *CA* cardiac arrest, *MAP* mean arterial pressure, *mmHg* millimetres of mercury

^*^Custom made mixture

All EPR models employed a flush-cooling protocol prior to the initiation of extracorporeal life support. Flush volumes and flow rates were remarkably consistent, with a median volume of 270 mL delivered at 50 mL/min. The temperature of the flushing solution varied between studies, ranging from ice-cold to room temperature, and several studies deliberately compared different temperature groups. Crystalloid solutions were used almost exclusively, with only a single study reporting a custom-made mixed solution. Target temperatures during the cardiac arrest and flush phase were uniformly low (median 15 °C), followed by controlled rewarming on ECLS to near-normothermia (median 34 °C).

The temporal structure of the EPR protocol showed considerable variability, particularly regarding the interval between flush completion and initiation of ECLS, which ranged from 14 to 75 min. In contrast, the duration of haemorrhagic shock prior to cardiac arrest was minimal in most studies, although one model reported a prolonged shock phase of up to 35 min. Importantly, EPR models did not use mean arterial pressure targets to guide reperfusion; instead, extracorporeal flow targets were predefined and used as the primary haemodynamic control variable during ECLS.

## Discussion

In this scoping review, we present the first comprehensive summary of existing rat models applying ECLS after global ischaemia. Our review revealed substantial methodological heterogeneity across models, with three primary experimental analogues to clinical scenarios: extracorporeal cardiopulmonary resuscitation (ECPR), emergency preservation and resuscitation (EPR), and deep hypothermic circulatory arrest (DHCA). These models differ markedly with respect to the method of ischaemia induction, experimental endpoints, and ECLS protocols, reflecting diverse research objectives. Furthermore, we noted considerable variability in the quality and completeness of reporting across studies. The implementation of standardized reporting guidelines would be a critical step toward enhancing comparability, transparency, and reproducibility, thereby accelerating the translational value of rat ECLS models.

Over the past two decades, global research activity in this field has grown substantially, although recent years suggest a plateau or slight decline—potentially influenced by external factors such as the COVID-19 pandemic [12, 25]. In addition, this apparent decline may reflect publication lag inherent to complex preclinical research, a gradual shift toward alternative experimental and clinical research approaches, or delays in publication. This trend underscores both the international scope and the increasing relevance of ECLS research. While large animal models have traditionally predominated the field, recent reviews emphasize the complementary value of small animal models, particularly rats [19, 31]. Compared to earlier reviews of CPR models, which reported a median group size of *n* = 10, our analysis revealed a higher median group size of 28 animals [31]. This suggests an increased level of statistical rigour and potentially enhanced translational relevance [16].

### Model characteristics and clinical alignment

In ECPR models, cardiac arrest was primarily induced by asphyxia (69%) or electrical stimulation (25%), reflecting clinical scenarios of hypoxia-induced or arrhythmogenic circulatory collapse. Here, next to cardiac and neurological outcomes, return of spontaneous circulation (ROSC) was defined as an experimental endpoint in 13 of 32 studies (41%), aligning with therapeutic priorities in the treatment of cardiac arrests. Of these, only 5 studies (38%) clearly defined ROSC. Although ROSC represents an essential prerequisite for survival, it was not consistently specified as an experimental goal. Notably, none of the studies implemented basic life support (BLS) or advanced life support (ALS) prior to the initiation of ECLS. This omission may limit the translational accuracy of the models, since BLS/ALS are integral components of resuscitation guidelines leading to a characteristic low-flow phase that contributes to reperfusion injury after whole-body ischaemia [6, 28]. Incorporating ALS measures prior to ECLS, as recommended in current clinical guidelines, should be considered to improve model realism and translational relevance [11, 26]. While chest compressions and defibrillation have already been demonstrated in rodent models, their application remains technically challenging due to anatomical constraints [33].

In contrast, EPR models of haemorrhagic arrest almost exclusively applied cardioplegia after pre-ischaemic haemorrhage (90%). DHCA models, on the other hand, predominantly induced ischaemia through hypothermia (95%), consistent with their use in planned circulatory arrest under controlled surgical conditions. EPR models most frequently assessed survival (90%) and neurological outcomes (100%), reflecting their focus on post-resuscitation recovery. ECPR studies evaluated neurological and cardiac outcomes equally (50% each), whereas survival was less commonly assessed (31%).

Clear differences were also observed in ECLS management strategies. Blood priming and transfusion were consistently applied in EPR models (100%), reflecting their design around haemorrhagic shock. In contrast, ECPR and DHCA models more commonly used balanced electrolyte or colloid solutions. Active cooling was a defining feature of DHCA models (100%), typically achieved through continuous cooling, while EPR models applied cooling with an intravascular flush immediately after cardiac arrest in 90% of cases. In comparison, active cooling was rarely used in ECPR studies (12%).

These methodological differences between ECPR, EPR, and DHCA models highlight the necessity of selecting experimental models that are appropriately aligned with the underlying pathophysiology and specific research objectives, as each model serves a distinct translational function within the heterogeneous field of ECLS research.

### Standardization and animal welfare

Continuous progress in cardiovascular small animal research relies on rigorous reporting of animal studies [2, 4]. Although the importance of standardized reporting in small animal models has been emphasized previously, adherence remains inconsistent [10, 31]. Beyond the heterogeneity of modelled clinical scenarios, our findings revealed that only 43 of the included studies (54%) reported baseline measurements prior to the induction of ischaemia, indicating a lack of standardized pre-intervention data. Similarly, randomization procedures were omitted in 26 studies (33%), and blinding was reported in only one, underscoring inconsistencies in methodological rigour.

While the consistent use of male animals contributes to experimental standardization, it raises concerns regarding translational applicability, given known sex-specific differences in outcomes to ischaemia and resuscitation [22, 34]. The underrepresentation of female animals limits the generalizability of findings and may obscure clinically relevant differences in treatment response. Incorporating both sexes in future ECLS models, or at minimum providing a clear justification for single-sex designs, would enhance translational relevance and align with current recommendations for sex-inclusive preclinical research [27].

25% of studies did not report a target extracorporeal flow rate on ECLS, and 91% failed to measure actual flow during the procedure. Where reported, the maximum extracorporeal flow varied widely between < 50 and 190 ml/kg/min, yet remained consistently below the physiological cardiac output of rats (322–384 ml/kg/min) [29]. Such discrepancies highlight potential mismatches between intended and delivered perfusion, which may alter physiological responses and compromise the translational validity.

The ECLS setup was relatively consistent across studies, typically employing roller or peristaltic pumps, custom-made or commercially available oxygenators, venous drainage via the jugular vein, and integration of a venous reservoir. Arterial return was most commonly achieved via the femoral or the caudal artery. Notably, components used in ECLS circuits—such as custom-made oxygenators—were often poorly described or not specified at all, limiting reproducibility and cross-study comparability.

The observed variability in endpoints reflects the diversity of rat ECLS models, highlighting the range of experimental designs tailored to different research questions. While 50 studies (63%) assessed neurological outcomes, relatively few incorporated functional or behavioural parameters, reflecting differing priorities in evaluating recovery and ECLS efficacy (see Table 4). This diversity in endpoints and methodologies illustrates how distinct models are optimized to address specific aspects of ischaemia and resuscitation research.

The quality and integrity of reported data, particularly concerning animal welfare, were frequently inadequate and warrant critical attention in future studies. Analgesia was not reported in 48 (61%) studies, often in conjunction with the use of neuromuscular blockers (62%) or inhalational anaesthesia alone (66%). Importantly, the *Guide for the Care and Use of Laboratory Animals* (National Research Council, US) emphasizes that inhalational anaesthesia by itself does not ensure sufficient analgesia [23]. Failure to report the analgesics used could mislead and raise significant concerns about adherence to established guidelines. Similarly, the single use of neuromuscular blockade was mentioned in some models; the lack of detailed reporting on adequate anaesthesia and analgesia is problematic and conflicts with the 3Rs principle. According to the Utstein-style guidelines for laboratory CPR research, animals receiving neuromuscular blockers must be unconscious and insensible to pain [15]. Consistent implementation and especially transparent reporting of peri- and postoperative pain management are essential to demonstrate adherence to ethical standards and ensure the reliability and reproducibility of experimental data [9, 27].

To improve the reproducibility of preclinical ECLS research, we developed a core dataset of minimum reporting items based on the data synthesis of this scoping review (see Supplement 9). The dataset builds on principles of good scientific practice and established reporting guidance (e.g., ARRIVE 2.0) and is complemented by ECLS- and scenario-specific parameters depending on the modelled setting (e.g., Utstein-style laboratory CPR reporting) [15, 27].

## Conclusion

Rat models of ECLS after global ischaemia provide an important platform for investigating clinically relevant scenarios, including ECPR, EPR, and DHCA. Current literature reveals considerable variability in methodological approaches and reporting qualities across the studies, highlighting the need to adapt existing animal reporting guidelines to small animal ECLS models to ensure transparency, comparability and reproducibility across studies.

Further research is needed to demonstrate the potential benefits of rat models compared to large animal models, especially in terms of mechanistic insights and feasibility for standardized preclinical studies.

## Materials and methods

A scoping review was conducted following the guideline for “Preferred Reporting Items for Systematic reviews and Meta-Analyses extension for Scoping Reviews” (PRISMA-ScR) [30]. A scoping review was chosen because rat ECLS models are highly heterogeneous in ischaemia induction, ECLS protocols, and outcome targets, which precludes meaningful meta-analysis. Our aim was to map the available evidence, summarize model characteristics and reporting practices, and identify key gaps to inform future study design and standardization.

The review was registered on Open Science Framework (OSF) Registries [8]. A protocol with a predefined search strategy, eligibility criteria, and methodological approach for data extraction was developed prior to the initiation of the review process. This protocol served to guide the systematic identification, selection, and analysis of relevant literature. Full details of the protocol are available in the supplemental material.

### Inclusion, exclusion criteria and definitions

All eligible articles published between January 2000 and December 2024 were included in the analysis. Studies were eligible if they involved a global ischaemia model followed by ECLS in rats. Global ischaemia was defined as complete loss of the body's own circulation that can but not necessarily be associated with CA.

ECLS was defined as the use of extracorporeal membrane oxygenation through an oxygenator to provide artificial oxygenation and gas exchange, in combination with the partial or complete replacement of the body’s circulatory system using an extracorporeal pump (including CPB-like circuits).

Non-primary literature, including reviews, commentaries or editorials, was excluded, and only studies with full-text availability were analysed. Only literature written in the English language was taken into account.

### Search strategy and screening

A systematic literature search was conducted on January 9, 2026, in PubMed, Web of Science (WOS) and Embase. The used search strategy, including search terms, is detailed in the online supplement (Supplement 3). All retrieved records were imported into reference management software (Zotero Version 7.0.2), where duplicate entries and retracted publications were identified and removed.

Two reviewers independently screened the titles and abstracts of the remaining records based on the predefined inclusion and exclusion criteria. Any discrepancies between reviewers or articles with unclear eligibility were resolved by including the record in the subsequent full-text screening phase. Inter-rater agreement was assessed using Cohen’s kappa statistic. In the final step, a single reviewer evaluated all eligible full-text articles for inclusion.

### Data extraction and analysis

Data were manually extracted from the final set of included full-text articles using a standardized data collection form. The extraction focused on key study characteristics, including animal model details, ischaemia protocols, ECLS setup, and outcome parameters. The selection of extracted variables was based on the Utstein-style guidelines for uniform reporting of laboratory CPR research [15], which, since 1996, has served as a framework for standardizing reported parameters in experimental resuscitation research. Where applicable, references to other articles were only considered for information on the technical setup of the ECLS system and not for primary data. Data visualization was performed using R statistical software (version 4.2.3).

## Limitations

Several limitations should be considered when interpreting the findings of this scoping review. First, our analysis included only English-language publications, which may introduce language bias and exclude relevant studies published in other languages. Second, many studies referred to prior work when describing their methods, leading to clustering within certain methodological frameworks or research groups. Third, the heterogeneity of study designs, endpoints, and outcome measures, while reflecting the diversity of rat ECLS models, complicates direct comparisons and meta-analytical synthesis. Despite these limitations, this review provides a comprehensive overview of the current landscape of rat ECLS models and identifies key areas for methodological improvement and standardization.

## Supplementary Information

Additional file 1.

## Data Availability

All analysed datasets are available from the corresponding author upon reasonable request.

## Abbreviations

CPR: Cardiopulmonary resuscitation
ECLS: Extracorporeal life support
CPB: Cardiopulmonary bypass
CA: Cardiac arrest
ECPR: Extracorporeal cardiopulmonary resuscitation
EPR: Emergency preservation and resuscitation
DHCA: Deep hypothermic circulatory arrest
ROSC: Return of spontaneous circulation
BLS: Basic life support
ALS: Advanced life support
IQR: Interquartile range
NSAID: Non-steroidal anti-inflammatory drugs
NMDA: N-Methyl-D-aspartate
