# Supplementary material for: Current rat models of extracorporeal life support following global ischaemia: a scoping review
Source: Intensive Care Med Exp. 2026 Feb 19;14:19. doi: 10.1186/s40635-026-00869-6 (PMC12920849; doi:10.1186/s40635-026-00869-6)
Supplement: Supplementary file 1 — Additional file 1. [file 40635_2026_869_MOESM1_ESM.docx]

**Supplemental Material**

**Supplement 1: Calculation of Cohens Kappa for inter-rater agreement in screening phase 1**

|  |  | Reviewer 2 | |  |  |
| --- | --- | --- | --- | --- | --- |
|  |  | 0 | 1 | SUM |  |
| Reviewer 1 | 0 | 633 | 25 | 658 | 0,2293578 |
|  | 1 | 30 | 84 | 114 | 0,7706422 |
|  | SUM | 663 | 109 | 772 |  |
|  |  | 0,85880829 | 0,14119171 |  |  |
|  |  |  |  |  |  |
|  |  |  |  |  |  |
|  | p0 | 0,92875648 |  |  |  |
|  | pe | 0,30578267 |  |  |  |
|  |  |  |  |  |  |
|  |  |  |  |  |  |
|  |  |  |  |  |  |
|  | Cohens Kappa: | 0,89737576 |  |  |  |

**Supplement 2: Syrcle protocol for animal intervention studies**

| 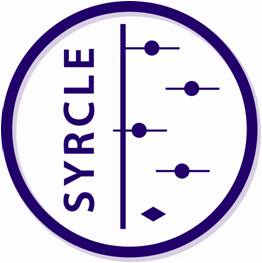 **Systematic Review Protocol for Animal Intervention Studies**  **Format by SYRCLE (**[**www.syrcle.nl**](http://www.syrcle.nl)**)**  **Version 2.0 (December 2014)** | | | | |
| --- | --- | --- | --- | --- |
| **Item #** | **Section/Subsection/Item** | **Description** | | **Check for approval** |
|  | A. General | | | |
| 1. | Title of the review | Current rat models of extracorporeal life support after global ischemia: A Scoping Review | | x |
| 2. | Authors (names, affiliations, contributions) | Dinkelaker J^1^, Pooth JS^2^, Trummer G^2^, Bröer S^3^, Busch HJ^2^, Schimmel M^4^, Wollborn J^5^, Brixius SJ^2^ | | x |
| 3. | Other contributors (names, affiliations, contributions) | - | | x |
| 4. | Contact person + e-mail address | Johannes Dinkelaker [Johannes.dinkelaker.cemt@uniklinik-freiburg.de](mailto:Johannes.dinkelaker.cemt@uniklinik-freiburg.de) | | X |
| 5. | Funding sources/sponsors | No funding | | X |
| 6. | Conflicts of interest | None | |  |
| 7. | Date and location of protocol registration | Open Science Framework (OSF) Registries 2025-05-13 | | X |
| 8. | Registration number (if applicable) | <https://doi.org/10.17605/OSF.IO/AXCFV> | | X |
| 9. | Stage of review at time of registration | Screening phase | | x |
|  | B. Objectives | | | |
|  | Background | | | |
| 10. | What is already known about this disease/model/intervention? Why is it important to do this review? | Rat models have been widely used in preclinical research due to their affordability, ease of handling, and well-characterized physiology. In recent years, various research groups have adapted rat models to study ECLS in the setting of global ischemia, employing different techniques, protocols, and outcome measures. However, no comprehensive overview currently exists to catalog and synthesize the scope, characteristics, and methodological diversity of these models. | | X |
|  | Research question | | | |
| 11. | Specify the disease/health problem of interest | Cardiac arrest, Resuscitation, Extracorporeal life support | | X |
| 12. | Specify the population/species studied | Rat | | X |
| 13. | Specify the intervention/exposure | CA or global ischemia AND ECLS | | X |
| 14. | Specify the control population | Experimental model of different categories | | X |
| 15. | Specify the outcome measures | Different outcomes | | X |
| 16. | State your research question (based on items 11-15) | How are rat models of global ischemia and extracorporeal resuscitation designed and reported in the literature from 2000 to 2024? | | X |
|  | C. Methods | | | |
|  | Search and study identification | | | |
| 17. | Identify literature databases to search (*e.g.* Pubmed, Embase, Web of science) | X MEDLINE via PubMed x Web of Science  □SCOPUS x EMBASE  □Other, namely:  □Specific journal(s), namely: | | x |
| 18. | Define electronic search strategies (*e.g.* use the [step by step search guide^15^](http://www.ncbi.nlm.nih.gov/pmc/articles/PMC3265183/pdf/LA-11-087.pdf) and animal search filters[^20,^](http://www.ncbi.nlm.nih.gov/pmc/articles/PMC3104815/pdf/LA-09-117.pdf) [^21^](http://lan.sagepub.com/content/48/1/88.full.pdf+html)) | When available, please add a supplementary file containing your search strategy: [supplement 1] | | x |
| 19. | Identify other sources for study identification | □Reference lists of included studies □Books  □Reference lists of relevant reviews  □Conference proceedings, namely:  □Contacting authors/ organisations, namely:  □Other, namely: | | x |
| 20. | Define search strategy for these other sources | - | | x |
|  | Study selection | | | |
| 21. | Define screening phases (*e.g.* pre-screening based on title/abstract, full text screening, both) | - 1. Pre-screening of title and abstract   2. Full text screening | | x |
| 22. | Specify (a) the number of reviewers per screening phase and (b) how discrepancies will be resolved | 1. Two reviewers 2. Include in full text screening | | x |
|  | *Define all inclusion and exclusion criteria based on:* | | | |
| 23. | Type of study (design) | Inclusion criteria: Primary research  Exclusion criteria: Reviews, editorials, no full text, retracted papers | | X |
| 24. | Type of animals/population (*e.g.* age, gender, disease model) | Inclusion criteria: Rat  Exclusion criteria: any other species | | X |
| 25. | Type of intervention (*e.g.* dosage, timing, frequency) | Inclusion criteria: Rat model; Cardiac arrest or global ischemia AND Extracorporeal life support  Exclusion criteria: No global ischemia | | X |
| 26. | Outcome measures | Inclusion criteria: -  Exclusion criteria: - | |  |
| 27. | Language restrictions | Inclusion criteria: English  Exclusion criteria: all other languages | | X |
| 28. | Publication date restrictions | Inclusion criteria: 01.01.2000 – 31.12.2024  Exclusion criteria: before 2000, after 2024 | | x |
| 29. | Other | Inclusion criteria: -  Exclusion criteria: Non-primary literature (reviews, commentary, editorials e.g.); No full text available | | x |
| 30. | Sort and prioritize your exclusion criteria per selection phase | Selection phase I:  1. Publication date  2. language  3. intervention  Selection phase II:  1. Intervention  2. Full text availability | | x |
|  | Study characteristics to be extracted (for assessment of external validity, reporting quality) | | | |
| 31. | Study ID (*e.g.* authors, year) | See record sheet | | X |
| 32. | Study design characteristics (*e.g.* experimental groups, number of animals) | See record sheet | | X |
| 33. | Animal model characteristics (*e.g.* species, gender, disease induction) | See record sheet | | X |
| 34. | Intervention characteristics (*e.g.* intervention, timing, duration) | See record sheet | | X |
| 35. | Outcome measures | See record sheet | | X |
| 36. | Other (*e.g.* drop-outs) | See record sheet | | X |
|  | Assessment risk of bias (internal validity) or study quality | | | |
| 37. | Specify (a) the number of reviewers assessing the risk of bias/study quality in each study and (b) how discrepancies will be resolved | Screening phase I: Two reviewers  Screening phase II: One reviewer  discrepancies between reviewers were resolved by including the record in the subsequent full-text screening phase | | x |
| 38. | Define criteria to assess (a) the internal validity of included studies (*e.g.* selection, performance, detection and attrition bias) and/or (b) other study quality measures (*e.g.* reporting quality, power) | □By use of [SYRCLE's Risk of Bias tool^4^](http://www.biomedcentral.com/1471-2288/14/43/abstract)  □By use of SYRCLE’s Risk of Bias tool, adapted as follows:  x By use of [CAMARADES' study quality checklist, e.g ^22^](http://www.ncbi.nlm.nih.gov/pubmed/15060322)  □By use of CAMARADES' study quality checklist, adapted as follows:  □Other criteria, namely: | | X |
|  | Collection of outcome data | | | |
| 39. | For each outcome measure, define the type of data to be extracted (*e.g.* continuous/dichotomous, unit of measurement) | See record sheet | | X |
| 40. | Methods for data extraction/retrieval (*e.g.* first extraction from graphs using a digital screen ruler, then contacting authors) | See record sheet | | X |
| 41. | Specify (a) the number of reviewers extracting data and (b) how discrepancies will be resolved | See record sheet | | X |
|  | Data analysis/synthesis | | | |
| 42. | Specify (per outcome measure) how you are planning to combine/compare the data (*e.g.* descriptive summary, meta-analysis) | Descriptive summary | | X |
| 43. | Specify (per outcome measure) how it will be decided whether a meta-analysis will be performed | - | | X |
|  | *If a meta-analysis seems feasible/sensible, specify (for each outcome measure):* | | | |
| 44. | The effect measure to be used (*e.g.* mean difference, standardized mean difference, risk ratio, odds ratio) | N/A | | X |
| 45. | The statistical model of analysis (*e.g.* random or fixed effects model) | N/A | | X |
| 46. | The statistical methods to assess heterogeneity (*e.g.* I^2^, Q) | N/A | | X |
| 47. | Which study characteristics will be examined as potential source of heterogeneity (subgroup analysis) | N/A | | X |
| 48. | Any sensitivity analyses you propose to perform | N/A | | X |
| 49. | Other details meta-analysis (*e.g.* correction for multiple testing, correction for multiple use of control group) | N/A | | X |
| 50. | The method for assessment of publication bias | N/A | | X |
|  | | | | |
| Final approval by (names, affiliations): | | JD | Date: | |

**Supplement 3: Search Strategy**

Search strategy Pubmed:

((ECMO[Tiab]) OR (Extracorporeal membrane oxygenation[Tiab]) OR (ECLS[Tiab]) OR (Extracorporeal live support[Tiab]) OR (E-CPR[Tiab]) OR (ECPR[Tiab]) OR (extracorpor* resus*[Tiab]) OR (CPB[Tiab]) OR (Cardiopulmonary bypass[Tiab]) OR (Heart lung machine[Tiab]) OR (Cardiopulmonary Bypass[MeSH Terms]) OR (Extracorporeal Membrane Oxygenation[MeSH Terms])) AND (Resus*[Tiab] OR CA[Tiab] OR cardiac arrest[Tiab] OR ischem*[Tiab] OR CPR[Tiab]) AND (Rat[Tiab] OR rats[Tiab] OR Rodent[Tiab] OR Rodentia[MeSH Terms])

Filter: 2000-2024

Search strategy Web of Science:

TS=(((ECMO) OR (Extracorporeal membrane oxygenation) OR (ECLS) OR (Extracorporeal live support) OR (E-CPR) OR (ECPR) OR (extracorpor* resus*) OR (CPB) OR (Cardiopulmonary bypass) OR (Heart lung machine) OR (Cardiopulmonary Bypass) OR (Extracorporeal Membrane Oxygenation)) AND (rat OR rats OR rodent) AND (Resus* OR CA OR cardiac arrest OR ischem* OR CPR))

Filter: 2000-2024

Search strategy Embase via OVID:

1 (ECMO or Extracorporeal membrane oxygenation or ECLS or Extracorporeal live support or E-CPR or ECPR or extracorpor* resus* or CPB or Cardiopulmonary bypass or Heart lung machine or Cardiopulmonary Bypass or Extracorporeal Membrane Oxygenation).mp. [mp=title, abstract, heading word, drug trade name, original title, device manufacturer, drug manufacturer, device trade name, keyword heading word, floating subheading word, candidate term word]

2 (rat or rats or rodent).mp. [mp=title, abstract, heading word, drug trade name, original title, device manufacturer, drug manufacturer, device trade name, keyword heading word, floating subheading word, candidate term word]

3 (Resus* or CA or cardiac arrest).mp. [mp=title, abstract, heading word, drug trade name, original title, device manufacturer, drug manufacturer, device trade name, keyword heading word, floating subheading word, candidate term word]

4 1 and 2

5 3 and 4

6 limit 5 to yr="2000 - 2024"

**Supplement 4: Alphabetic list of included articles**

1. Ahmed, N. et al. Fingolimod Plays Role in Attenuation of Myocardial Injury Related to Experimental Model of Cardiac Arrest and Extracorporeal Life Support Resuscitation. INTERNATIONAL JOURNAL OF MOLECULAR SCIENCES 20, (2019).

2. Ahmed, N. et al. Cardioprotective Effects of Sphingosine-1-Phosphate Receptor Immunomodulator FTY720 in a Clinically Relevant Model of Cardioplegic Arrest and Cardiopulmonary Bypass. FRONTIERS IN PHARMACOLOGY 10, (2019).

3. Ali, A. A. et al. Rat model of veno-arterial extracorporeal membrane oxygenation. JOURNAL OF TRANSLATIONAL MEDICINE 12, (2014).

4. Bartels, K. et al. Effects of Deep Hypothermic Circulatory Arrest on the Blood Brain Barrier in a Cardiopulmonary Bypass Model - A Pilot Study. HEART LUNG AND CIRCULATION 23, 981–984 (2014).

5. Boller, M. et al. A combination of metabolic strategies plus cardiopulmonary bypass improves short-term resuscitation from prolonged lethal cardiac arrest. Resuscitation 82 Suppl 2, S27-34 (2011).

6. Chang, R.-W., Hsu, M.-C., Lee, T.-S., Chen, Y.-S. & Wang, C.-H. Selective brain perfusion improves the neurological outcomes after extracorporeal cardiopulmonary resuscitation in a rat model. ARTIFICIAL ORGANS 48, 743–752 (2024).

7. Chang, R.-W., Luo, C.-M., Yu, H.-Y., Chen, Y.-S. & Wang, C.-H. Investigation of the pathophysiology of cardiopulmonary bypass using rodent extracorporeal life support model. BMC CARDIOVASCULAR DISORDERS 17, (2017).

8. Chen, Q., Lei, Y.-Q., Liu, J.-F., Wang, Z.-C. & Cao, H. Triptolide improves neurobehavioral functions, inflammation, and oxidative stress in rats under deep hypothermic circulatory arrest. AGING-US 13, 3031–3044 (2021).

9. Chen, Q., Lei, Y.-Q., Liu, J.-F., Wang, Z.-C. & Cao, H. Beneficial effects of chlorogenic acid treatment on neuroinflammation after deep hypothermic circulatory arrest may be mediated through CYLD/NF-κB signaling. BRAIN RESEARCH 1767, (2021).

10. Chen, Q., Sun, K.-P., Huang, J.-S., Wang, Z.-C. & Hong, Z.-N. Resveratrol attenuates neuroinflammation after deep hypothermia with circulatory arrest in rats. BRAIN RESEARCH BULLETIN 155, 145–154 (2020).

11. Chen, S., Yu, J., Xue, P., Hei, F. & Guan, Y. EXTRACORPOREAL CARDIOPULMONARY RESUSCITATION WITH THERAPEUTIC HYPOTHERMIA MITIGATES KIDNEY INJURY AFTER CARDIAC ARREST IN RATS. SHOCK 60, 315–324 (2023).

12. Choi, J. et al. Tissue-Specific Metabolic Profiles After Prolonged Cardiac Arrest Reveal Brain Metabolome Dysfunction Predominantly After Resuscitation. JOURNAL OF THE AMERICAN HEART ASSOCIATION 8, (2019).

13. Choudhary, R. C. et al. Multi-drug Cocktail Approach After Asphyxial Cardiac Arrest And Cardiopulmonary Bypass Resuscitation In Rats. CIRCULATION 146, (2022).

14. Dian-San, S., Xiang-Rui, W., Yongjun, Z. & Yan-Hua, Z. Low hematocrit worsens cerebral injury after prolonged hypothermic circulatory arrest in rats. CANADIAN JOURNAL OF ANAESTHESIA-JOURNAL CANADIEN D ANESTHESIE 53, 1220–1229 (2006).

15. Drabek, T. et al. Prolonged deep hypothermic circulatory arrest in rats can be achieved without cognitive deficits. LIFE SCIENCES 81, 543–552 (2007).

16. Drabek, T. et al. Assessment of the delta opioid agonist DADLE in a rat model of lethal hemorrhage treated by emergency preservation and resuscitation. RESUSCITATION 77, 220–228 (2008).

17. Drabek, T. et al. Microglial depletion using intrahippocampal injection of liposome-encapsulated clodronate in prolonged hypothermic cardiac arrest in rats. RESUSCITATION 83, 517–526 (2012).

18. Drabek, T. et al. Minocycline attenuates brain tissue levels of TNF-α produced by neurons after prolonged hypothermic cardiac arrest in rats. RESUSCITATION 85, 284–291 (2014).

19. Drabek, T. et al. Emergency preservation and delayed resuscitation allows normal recovery after exsanguination cardiac arrest in rats: A feasibility trial. CRITICAL CARE MEDICINE 35, 532–537 (2007).

20. Drabek, T. et al. Exsanguination cardiac arrest in rats treated by 60 min, but not 75 min, emergency preservation and delayed resuscitation is associated with intact outcome. RESUSCITATION 75, 114–123 (2007).

21. Drabek, T. et al. Deep Hypothermia Attenuates Microglial Proliferation Independent of Neuronal Death After Prolonged Cardiac Arrest in Rats. ANESTHESIA AND ANALGESIA 109, 914–923 (2009).

22. Drabek, T. et al. Unique Brain Region-Dependent Cytokine Signatures After Prolonged Hypothermic Cardiac Arrest in Rats. THERAPEUTIC HYPOTHERMIA AND TEMPERATURE MANAGEMENT 5, 26–39 (2015).

23. Engels, M. et al. A cardiopulmonary bypass with deep hypothermic circulatory arrest rat model for the investigation of the systemic inflammation response and induced organ damage. JOURNAL OF INFLAMMATION-LONDON 11, (2014).

24. Gao, Y. et al. Moderate Hypothermic Circulatory Arrest Model in Rats: A New Model with Hyperkalemia-Induced Cardioplegia. HEART SURGERY FORUM 25, E548–E552 (2022).

25. Gordan, M. L. et al. Fast rewarming after deep hypothermic circulatory arrest in rats impairs histologic outcome and increases NFκB expression in the brain. PERFUSION-UK 25, 349–354 (2010).

26. Han, F. et al. A rodent model of emergency cardiopulmonary bypass resuscitation with different temperatures after asphyxial cardiac arrest. RESUSCITATION 81, 93–99 (2010).

27. Han, F. et al. Protein nitration and poly-ADP-ribosylation in brain after rapid exsanguination cardiac arrest in a rat model of emergency preservation and resuscitation. RESUSCITATION 79, 301–310 (2008).

28. Janata, A. et al. Extracorporeal Versus Conventional Cardiopulmonary Resuscitation After Ventricular Fibrillation Cardiac Arrest in Rats: A Feasibility Trial. CRITICAL CARE MEDICINE 41, E211–E222 (2013).

29. Jenke, A. et al. AdipoRon Attenuates Inflammation and Impairment of Cardiac Function Associated With Cardiopulmonary Bypass-Induced Systemic Inflammatory Response Syndrome. JOURNAL OF THE AMERICAN HEART ASSOCIATION 10, (2021).

30. Jiang, X. et al. A novel augmented venous-drainage model of cardiopulmonary bypass for deep hypothermic circulatory arrest without blood priming. PERFUSION-UK 33, 297–302 (2018).

31. Jiang, X. et al. Protection of the rat brain from hypothermic circulatory arrest injury by a chipmunk protein. JOURNAL OF THORACIC AND CARDIOVASCULAR SURGERY 156, 525–536 (2018).

32. Jungwirth, B. et al. Neurologic outcome after cardiopulmonary bypass with deep hypothermic circulatory arrest in rats: Description of a new model. JOURNAL OF THORACIC AND CARDIOVASCULAR SURGERY 131, 805–812 (2006).

33. Karhausen, J. et al. Intestinal Mast Cells Mediate Gut Injury and Systemic Inflammation in a Rat Model of Deep Hypothermic Circulatory Arrest. CRITICAL CARE MEDICINE 41, E200–E210 (2013).

34. Kellermann, K. et al. Perioperative moxifloxacin treatment in rats subjected to deep hypothermic circulatory arrest: Reduction in cerebral inflammation but without improvement in cognitive performance. JOURNAL OF THORACIC AND CARDIOVASCULAR SURGERY 141, 796–802 (2011).

35. Kellermann, K. et al. Functional Outcome in Female Rats after 45 Minutes of Deep Hypothermic Circulatory Arrest: Gender Matters. THORACIC AND CARDIOVASCULAR SURGEON 61, 52–65 (2013).

36. Kellermann, K. et al. Long-term assessment of NFκB expression in the brain and neurologic outcome following deep hypothermic circulatory arrest in rats. PERFUSION-UK 24, 429–436 (2009).

37. Kim, J., Lampe, J. W., Yin, T., Shinozaki, K. & Becker, L. B. Phospholipid alterations in the brain and heart in a rat model of asphyxia-induced cardiac arrest and cardiopulmonary bypass resuscitation. MOLECULAR AND CELLULAR BIOCHEMISTRY 408, 273–281 (2015).

38. Kim, J. et al. The Responses of Tissues from the Brain, Heart, Kidney, and Liver to Resuscitation following Prolonged Cardiac Arrest by Examining Mitochondrial Respiration in Rats. OXIDATIVE MEDICINE AND CELLULAR LONGEVITY 2016, (2016).

39. Kim, J., Yin, T., Shinozaki, K., Lampe, J. W. & Becker, L. B. DHA-supplemented diet increases the survival of rats following asphyxia-induced cardiac arrest and cardiopulmonary bypass resuscitation. SCIENTIFIC REPORTS 6, (2016).

40. Kim, J., Yin, T., Shinozaki, K., Lampe, J. W. & Becker, L. B. Potential of lysophosphatidylinositol as a prognostic indicator of cardiac arrest using a rat model. BIOMARKERS 22, 755–763 (2017).

41. Kim, J. et al. Examination of Physiological Function and Biochemical Disorders in a Rat Model of Prolonged Asphyxia-Induced Cardiac Arrest followed by Cardio Pulmonary Bypass Resuscitation. PLOS ONE 9, (2014).

42. Lahoud-Rahme, M. S. et al. Blood-brain barrier integrity in a rat model of emergency preservation and resuscitation. RESUSCITATION 80, 484–488 (2009).

43. Li, J. et al. Article Bradykinin induces acute kidney injury after hypothermic circulatory arrest through the repression of the Nrf2-xCT pathway. ISCIENCE 27, (2024).

44. Li, Y.-A. et al. Differential expression profiles of circular RNAs in the rat hippocampus after deep hypothermic circulatory arrest. ARTIFICIAL ORGANS 45, 866–880 (2021).

45. Linardi, D. et al. Slow versus fast rewarming after hypothermic circulatory arrest: effects on neuroinflammation and cerebral oedema. EUROPEAN JOURNAL OF CARDIO-THORACIC SURGERY 58, 792–800 (2020).

46. Liu, M. et al. A novel target to reduce microglial inflammation and neuronal damage after deep hypothermic circulatory arrest. The Journal of Thoracic and Cardiovascular Surgery 159, 2431-2444.e7 (2020).

47. Liu, M., Zeng, Q., Li, Y., Liu, G. & Ji, B. Neurologic recovery after deep hypothermic circulatory arrest in rats: A description of a long-term survival model without blood priming. ARTIFICIAL ORGANS 43, 551–560 (2019).

48. Ma, Q. et al. Annexin A1 Bioactive Peptide Promotes Resolution of Neuroinflammation in a Rat Model of Exsanguinating Cardiac Arrest Treated by Emergency Preservation and Resuscitation. FRONTIERS IN NEUROSCIENCE 13, (2019).

49. Magnet, I. A. M. et al. EXTRACORPOREAL LIFE SUPPORT INCREASES SURVIVAL AFTER PROLONGED VENTRICULAR FIBRILLATION CARDIAC ARREST IN THE RAT. SHOCK 48, 674–680 (2017).

50. Magnet, I. et al. Neuroprotection with hypothermic reperfusion and extracorporeal cardiopulmonary resuscitation - A randomized controlled animal trial of prolonged ventricular fibrillation cardiac arrest in rats. JOURNAL OF CEREBRAL BLOOD FLOW AND METABOLISM (2024) doi:10.1177/0271678X241281485.

51. Mao, Q. et al. Remimazolam ameliorates postoperative cognitive dysfunction after deep hypothermic circulatory arrest through HMGB1-TLR4-NF-κB pathway. Brain Research Bulletin 217, 111086 (2024).

52. Pinto, A. et al. The extracellular isoform of superoxide dismutase has a significant impact on cardiovascular ischaemia and reperfusion injury during cardiopulmonary bypass. EUROPEAN JOURNAL OF CARDIO-THORACIC SURGERY 50, 1035–1044 (2016).

53. Pinto, A. et al. Modulation of Immunologic Response by Preventive Everolimus Application in a Rat CPB Model. INFLAMMATION 39, 1771–1782 (2016).

54. Rungatscher, A. et al. Role of calcium desensitization in the treatment of myocardial dysfunction after deep hypothermic circulatory arrest. CRITICAL CARE 17, (2013).

55. Rungatscher, A. et al. Cardioprotective effect of δ-opioid receptor agonist vs mild therapeutic hypothermia in a rat model of cardiac arrest with extracorporeal life support. RESUSCITATION 84, 244–248 (2013).

56. Rungatscher, A. et al. Levosimendan is superior to epinephrine in improving myocardial function after cardiopulmonary bypass with deep hypothermic circulatory arrest in rats. JOURNAL OF THORACIC AND CARDIOVASCULAR SURGERY 143, 209–214 (2012).

57. Schanche, T., Kondratiev, T. & Tveita, T. Extracorporeal rewarming from experimental hypothermia: Effects of hydroxyethyl starch versus saline priming on fluid balance and blood flow distribution. EXPERIMENTAL PHYSIOLOGY 104, 1353–1362 (2019).

58. Schober, A. et al. Microdialysis Assessment of Cerebral Perfusion during Cardiac Arrest, Extracorporeal Life Support and Cardiopulmonary Resuscitation in Rats - A Pilot Trial. PLOS ONE 11, (2016).

59. Shim, J.-K., Ma, Q., Zhang, Z., Podgoreanu, M. V. & Mackensen, G. B. Effect of pregabalin on cerebral outcome after cardiopulmonary bypass with deep hypothermic circulatory arrest in rats. JOURNAL OF THORACIC AND CARDIOVASCULAR SURGERY 148, 298–303 (2014).

60. Shinozaki, K. et al. The effects of early high-volume hemofiltration on prolonged cardiac arrest in rats with reperfusion by cardiopulmonary bypass: a randomized controlled animal study. Intensive Care Med Exp 4, 25 (2016).

61. Shinozaki, K. et al. Developing dual hemofiltration plus cardiopulmonary bypass in rodents. JOURNAL OF SURGICAL RESEARCH 195, 196–203 (2015).

62. Shoaib, M. et al. Plasma metabolomics supports the use of long-duration cardiac arrest rodent model to study human disease by demonstrating similar metabolic alterations. SCIENTIFIC REPORTS 10, (2020).

63. Steinbrenner, H. et al. Selenium Pretreatment for Mitigation of Ischemia/Reperfusion Injury in Cardiovascular Surgery: Influence on Acute Organ Damage and Inflammatory Response. INFLAMMATION 39, 1363–1376 (2016).

64. Stommel, A.-M. et al. A ventricular fibrillation cardiac arrest model with extracorporeal cardiopulmonary resuscitation in rats: 8 minutes arrest time leads to increased myocardial damage but does not increase neuronal damage compared to 6 minutes. FRONTIERS IN VETERINARY SCIENCE 10, (2023).

65. Su, D., Wang, X., Zheng, Y., Zhao, Y. & Zhang, T. Retrograde cerebral perfusion of oxygenated, compacted red blood cells attenuates brain damage after hypothermia circulation arrest of rat. ACTA ANAESTHESIOLOGICA SCANDINAVICA 49, 1172–1181 (2005).

66. Tam, J. et al. The role of decreased cardiolipin and impaired electron transport chain in brain damage due to cardiac arrest. NEUROCHEMISTRY INTERNATIONAL 120, 200–205 (2018).

67. Wang, Y. et al. Inhibition of microRNA-29c protects the brain in a rat model of prolonged hypothermic circulatory arrest. JOURNAL OF THORACIC AND CARDIOVASCULAR SURGERY 150, 675–683 (2015).

68. Warenits, A.-M. et al. REDUCTION OF SERIOUS ADVERSE EVENTS DEMANDING STUDY EXCLUSION IN MODEL DEVELOPMENT: EXTRACORPOREAL LIFE SUPPORT RESUSCITATION OF VENTRICULAR FIBRILLATION CARDIAC ARREST IN RATS. SHOCK 46, 704–712 (2016).

69. Wollborn, J. et al. Phosphodiesterase-4 inhibition reduces ECLS-induced vascular permeability and improves microcirculation in a rodent model of extracorporeal resuscitation. AMERICAN JOURNAL OF PHYSIOLOGY-HEART AND CIRCULATORY PHYSIOLOGY 316, H751–H761 (2019).

70. Woods, C. E. et al. In Vivo Post-Cardiac Arrest Myocardial Dysfunction Is Supported by Ca<SUP>2+</SUP>/Calmodulin-Dependent Protein Kinase II-Mediated Calcium Long-Term Potentiation and Mitigated by Alda-1, an Agonist of Aldehyde Dehydrogenase Type 2. CIRCULATION 134, 961+ (2016).

71. Xiong, Y., Zheng, Q., Wang, C., Yan, W. & He, W. Organ damage evaluation in a temperature-controlled circulatory arrest rat model. BMC CARDIOVASCULAR DISORDERS 22, (2022).

72. Yeh, S. T., Aune, S. E., Wilgus, T. A., Parent, A. E. & Angelos, M. G. Hyperoxemic reperfusion after prolonged cardiac arrest in a rat cardiopulmonary bypass resuscitation model. RESUSCITATION 84, 114–120 (2013).

73. Yin, T. et al. Hydrogen gas with extracorporeal cardiopulmonary resuscitation improves survival after prolonged cardiac arrest in rats. JOURNAL OF TRANSLATIONAL MEDICINE 19, (2021).

74. Yu, M.-H. et al. Cannabinoid Receptor Agonist WIN55, 212-2 Attenuates Injury in the Hippocampus of Rats after Deep Hypothermic Circulatory Arrest. BRAIN SCIENCES 13, (2023).

75. Yuan, L. et al. Cerebral Blood Flow Changes during Rat Cardiopulmonary Bypass and Deep Hypothermic Circulatory Arrest Model: A Preliminary Study. in 2013 35TH ANNUAL INTERNATIONAL CONFERENCE OF THE IEEE ENGINEERING IN MEDICINE AND BIOLOGY SOCIETY (EMBC) 1807–1810 (IEEE Engn Med Biol Soc; Japanese Soc Med & Biol Engn, 2013).

76. Zhai, K. et al. Neuroprotective effect of selective hypothermic cerebral perfusion in extracorporeal cardiopulmonary resuscitation: A preclinical study. JTCVS OPEN 12, 221–233 (2022).

77. Zhang, W. et al. Increased Survival Time With SS-31 After Prolonged Cardiac Arrest in Rats. HEART LUNG AND CIRCULATION 28, 505–508 (2019).

78. Zhang, Z. et al. Neurprotective Effects of Annexin A1 Tripeptide after Deep Hypothermic Circulatry Arrest in Rates. FRONTIERS IN IMMUNOLOGY 8, (2017).

79. Zhu, M., Zhao, Y., Zheng, Y., Su, D. & Wang, X. Relative Higher Hematocrit Attenuates the Cerebral Excitatory Amino Acid Elevation Induced by Deep Hypothermic Circulatory Arrest in Rats. THERAPEUTIC HYPOTHERMIA AND TEMPERATURE MANAGEMENT 3, 140–142 (2013).

**Supplement 5: Comparative Animal Characteristics**

| **Table 1.** Animal characteristics and group composition | | |  |  |  |
| --- | --- | --- | --- | --- | --- |
|  |  |  |  |  |  |
| Variable | | All studies (n = 79) | Extracorporeal cardiopulmonary resuscitation (ECPR) (n = 32) | Emergency preservation and resuscitation (EPR)* (n = 10) | Deep hypothermic circulatory arrest (DHCA) (n = 37) |
|  |  |  |  |  |  |
| **Animal** | |  |  |  |  |
| Number of animals - median (quartiles) | | 28 (18, 34) | 26 (18, 33) | 26 (18, 32) | 30 (18, 40) |
|  |  |  |  |  |  |
| Species - n (%) | |  |  |  |  |
|  | Sprague Dawley | 65 (82) | 28 (88) | 10 (100) | 27 (73) |
|  | Wistar | 12 (15) | 3 (9) | 0 (0) | 9 (24) |
|  | Other or not reported | 2 (3) | 1 (3) | 0 (0) | 1 (3) |
|  |  |  |  |  |  |
| Sex - n (%) | |  |  |  |  |
|  | Male | 76 (96) | 31 (97) | 10 (100) | 35 (95) |
|  | Female | 0 (0) | 0 (0) | 0 (0) | 0 (0) |
|  | Both | 1 (1) | 0 (0) | 0 (0) | 1 (3) |
|  | Not reported | 2 (3) | 1 (3) | 0 (0) | 1 (3) |
|  |  |  |  |  |  |
| Age [weeks] - n (%) | |  |  |  |  |
|  | <10 | 5 (6) | 2 (6) | 0 (0) | 3 (8) |
|  | 10-12 | 5 (6) | 1 (3) | 0 (0) | 4 (11) |
|  | 12-14 | 8 (10) | 0 (0) | 1 (10) | 7 (19) |
|  | 14-16 | 2 (3) | 0 (0) | 0 (0) | 2 (5) |
|  | 16-18 | 2 (3) | 0 (0) | 0 (0) | 2 (5) |
|  | >18 | 0 (0) | 0 (0) | 0 (0) | 0 (0) |
|  | not reported | 57 (72) | 29 (91) | 9 (90) | 19 (51) |
|  |  |  |  |  |  |
| Weight [g] - median (quartiles) | |  |  |  |  |
|  | Min | 400 (350, 445) | 415 (350, 450) | 350 (350, 350) | 400 (350, 402) |
|  | Max | 450 (400, 500) | 478 (350, 530) | 400 (350, 400) | 450 (350, 500) |
|  |  |  |  |  |  |
| Morbidity - n (%) | |  |  |  |  |
|  | Normal animals | 76 (96) | 31 (97) | 10 (100) | 35 (95) |
|  | Genetically modified | 2 (3) | 0 (0) | 0 (0) | 2 (5) |
|  | Not reported | 1 (1) | 0 (0) | 0 (0) | 0 (0) |
|  |  |  |  |  |  |
| **Groups** | |  |  |  |  |
|  | Number of groups - median (quartiles) | 3 (2, 4) | 3 (2, 3) | 4 (3, 4) | 3 (2, 4) |
|  | Number of animals in smallest intervention group - median (quartiles) | 8 (6, 10) | 8 (6, 11) | 6 (6, 7) | 8 (6, 10) |
|  | Animals with global ischemia and ECLS - median (quartiles) | 18 (10, 30) | 16 (8, 27) | 18 (14, 29) | 20 (12, 30) |
|  |  |  |  |  |  |
| SHAM Group - n (%) | |  |  |  |  |
|  | No/not reported | 28 (35) | 15 (47) | 4 (40) | 9 (24) |
|  | Yes | 51 (65) | 17 (53) | 6 (60) | 28 (76) |
|  |  |  |  |  |  |
| Randomisation* - n (%) | |  |  |  |  |
|  | No/not reported | 26 (33) | 12 (38) | 6 (60) | 8 (22) |
|  | Yes | 53 (67) | 20 (63) | 4 (40) | 29 (78) |
|  |  |  |  |  |  |
| Blinding - n (%) | |  |  |  |  |
|  | No/not reported | 78 (99) | 31 (97) | 10 (100) | 37 (100) |
|  | Yes | 1 (1) | 1 (3) | 0 (0) | 0 (0) |
|  |  |  |  |  |  |
| **Not applicable in 5 studies because only one group was used; n, number; Min, Minimum; Max, Maximum* | | | | | |

**Supplement 6: Comparative Baseline Characteristics**

| **Table 2.** Baseline characteristics | |  |  |  |  |
| --- | --- | --- | --- | --- | --- |
|  |  |  |  |  |  |
| Variable | | All studies (n = 79) | Extracorporeal cardiopulmonary resuscitation (ECPR) (n = 32) | Emergency preservation and resuscitation (EPR)* (n = 10) | Deep hypothermic circulatory arrest (DHCA) (n = 37) |
|  |  |  |  |  |  |
| Animals fasted - n (%) | |  |  |  |  |
|  | No/not reported | 66 (84) | 31 (97) | 9 (90) | 26 (70) |
|  | Yes | 13 (16) | 1 (3) | 1 (10) | 11 (30) |
|  |  |  |  |  |  |
| Anaesthesia - n (%) | | 79 (100) |  |  |  |
|  | Inhalation | 52 (66) | 25 (78) | 10 (100) | 17 (46) |
|  | Inhalation and intraperitoneal | 11 (14) | 2 (6) | 0 (0) | 9 (24) |
|  | Inhalation and intravenous | 8 (10) | 2 (6) | 0 (0) | 6 (16) |
|  | Intraperitoneal | 4 (5) | 2 (6) | 0 (0) | 2 (5) |
|  | Intravenous and intraperitoneal | 1 (1) | 1 (3) | 0 (0) | 0 (0) |
|  | Not reported | 3 (4) | 0 (0) | 0 (0) | 3 (8) |
|  |  |  |  |  |  |
| Pain medication - n (%) | |  |  |  |  |
|  | No/not reported | 48 (61) | 21 (66) | 10 (100) | 17 (46) |
|  | Yes | 31 (39) | 11 (34) | 0 (0) | 20 (54) |
|  |  |  |  |  |  |
|  | Fentanyl | 18 (58) | 1 (9) | N/A | 17 (85) |
|  | Buprenorphine | 6 (19) | 5 (45) | N/A | 1 (5) |
|  | Ketoprofen | 2 (6) | 1 (9) | N/A | 1 (5) |
|  | Ketamine | 1 (3) | 1 (9) | N/A | 0 (0) |
|  | Fentanyl and ketamine | 1 (3) | 0 (0) | N/A | 1 (5) |
|  | Piritramide | 1 (3) | 1 (9) | N/A | 0 (0) |
|  | Piritramide and carprofen | 1 (3) | 1 (9) | N/A | 0 (0) |
|  | Not reported | 1 (3) | 1 (9) | N/A | 0 (0) |
|  |  |  |  |  |  |
| Muscle relaxation - n (%) | |  |  |  |  |
|  | No/not reported | 49 (62) | 11 (34) | 9 (90) | 29 (78) |
|  | Yes | 30 (38) | 21 (66) | 1 (10) | 8 (22) |
|  |  |  |  |  |  |
|  | Relaxation without reported pain medication | 19 (63) | 16 (76) | 1 (100) | 2 (25) |
|  |  |  |  |  |  |
| Monitoring - n (%) | |  |  |  |  |
|  | Arterial blood pressure | 76 (96) | 31 (97) | 10 (100) | 35 (95) |
|  | Temperature | 73 (92) | 27 (84) | 10 (100) | 36 (97) |
|  | Blood gas analysis | 72 (91) | 29 (91) | 10 (100) | 33 (89) |
|  | Electrocardiography | 53 (67) | 22 (69) | 10 (100) | 21 (57) |
|  | Central venous pressure | 20 (25) | 10 (31) | 9 (90) | 1 (3) |
|  | Pulse oximetry | 10 (13) | 2 (6) | 0 (0) | 8 (22) |
|  | Cardiac output | 5 (6) | 5 (16) | 0 (0) | 0 (0) |
|  | Electroencephalography | 1 (1) | 1 (3) | 0 (0) | 0 (0) |
|  | Somatosensory evoked potentials | 0 (0) | 0 (0) | 0 (0) | 0 (0) |
|  |  |  |  |  |  |
| Baseline measurements before ischemia - n (%) | | |  |  |  |
|  | Yes | 43 (54) | 23 (72) | 8 (80) | 12 (32) |
|  | No/not reported | 36 (46) | 9 (28) | 2 (20) | 25 (68) |
|  |  |  |  |  |  |
|  | *n, number;* |  |  |  |  |

**Supplement 7: Comparative Model Characteristics**

| **Table 3.** Model characteristics | |  |  |  |  |
| --- | --- | --- | --- | --- | --- |
|  |  |  |  |  |  |
| Variable |  | All studies (n = 79) | ECPR (n = 32) | EPR* (n = 10) | DHCA (n = 37) |
|  |  |  |  |  |  |
| **Technical specifications of extracorporeal life support (ECLS)** | | | |  |  |
| Cannulation sites for ECLS - n (%) | |  |  |  |  |
| Art. | Art. Femoralis | 42 (53) | 27 (84) | 9 (90) | 6 (16) |
|  | Art. Carotis communis | 9 (11) | 4 (13) | 0 (0) | 5 (14) |
|  | Tail or caudal artery | 28 (35) | 1 (3) | 1 (10) | 26 (70) |
|  |  |  |  |  |  |
| Ven. | Ven. Jugularis | 76 (96) | 31 (97) | 10 (100) | 35 (95) |
|  | Right atrium | 3 (4) | 1 (3) | 0 (0) | 2 (5) |
|  |  |  |  |  |  |
| Venous reservoir - n (%) | |  |  |  |  |
|  | Yes | 70 (89) | 29 (91) | 10 (100) | 31 (84) |
|  | No/not reported | 9 (11) | 3 (9) | 0 (0) | 6 (16) |
|  |  |  |  |  |  |
| Type of pump used - n (%) | |  |  |  |  |
|  | Roller pump | 56 (71) | 29 (91) | 9 (90) | 18 (49) |
|  | Peristaltic pump | 21 (27) | 3 (9) | 1 (10) | 17 (46) |
|  | Other/not specified | 2 (3) | 0 (0) | 0 (0) | 2 (5) |
|  |  |  |  |  |  |
| Used oxygenator - n (%) | |  |  |  |  |
|  | Custom made | 32 (41) | 3 (9) | 9 (90) | 20 (54) |
|  | Commercially available | 25 (32) | 17 (53) | 1 (10) | 7 (19) |
|  | Not specified | 22 (28) | 12 (38) | 0 (0) | 10 (27) |
|  |  |  |  |  |  |
| Extracorporeal flow measured - n (%) | |  |  |  |  |
|  | Yes | 7 (9) | 2 (6) | 0 (0) | 5 (14) |
|  | No/not reported | 72 (91) | 30 (94) | 10 (100) | 32 (86) |
|  |  |  |  |  |  |
| ** All models performed haemorrhage before induction of ischemia; Art., arterial; Ven., venous; CA, Cardiac arrest; ECPR, Extracorporeal cardiopulmonary resuscitation; EPR, Emergency preservation and resuscitation; ROSC, Return of spontaneous circulation; ECLS, Extracorporeal life support* | | | | | |

**Supplement 8: Comparative characteristics of experimental procedures**

| **Table 4. Comparative characteristics of experimental procedures** | | | |  |  |
| --- | --- | --- | --- | --- | --- |
| Variable | | All studies (n = 37)) | ECPR (n = 32) | EPR* (n = 10) | DHCA (n = 37) |
|  |  |  |  |  |  |
| **Induction and ischemia** | |  |  |  |  |
|  |  |  |  |  |  |
| FiO2 before CA - % (quartiles) | | 50 (30, 95) | 50 (30, 93) | 25 (25, 25) | 50 (45, 100) |
|  |  |  |  |  |  |
| Technique of induction of global ischemia - n (%) | | |  |  |  |
|  | Hypothermia | 35 (44) | 0 (0) | 0 (0) | 35 (95) |
|  | Asphyxia | 22 (28) | 22 (69) | 0 (0) | 0 (0) |
|  | Electric | 9 (11) | 8 (25) | 1 (10) | 0 (0) |
|  | Cadioplegia | 13 (16) | 2 (6) | 9 (90) | 2 (5) |
|  |  |  |  |  |  |
| Clear definition of ischemia - n (%) | |  |  |  |  |
|  | Yes | 36 (46) | 20 (63) | 1 (10) | 15 (41) |
|  | No | 43 (54) | 12 (38) | 9 (90) | 22 (59) |
|  |  |  |  |  |  |
| Maximum no flow time [min] | |  |  |  |  |
|  | median (quartiles) | 30 (15, 45) | 12 (8, 20) | 20 (20, 75) | 45 (41, 60) |
|  | Minimum - maximum | 1 - 105 | 4 - 30 | 1 - 80 | 1 - 105 |
|  |  |  |  |  |  |
|  |  |  |  |  |  |
| **Extracorporeal circulation** | |  |  |  |  |
|  |  |  |  |  |  |
| Maximum Duration of ECLS [min] | |  |  |  |  |
|  | Median (quartiles) | 60 (30, 60) | 30 (30, 60) | 60 (60, 60) | 60 (30, 90) |
|  | Minimum - maximum | 10 - 180 | 10 - 180 | 60 - 80 | 30 - 120 |
|  |  |  |  |  |  |
| Type of priming - n (%) | |  |  |  |  |
|  | Balanced electrolyte solution | 17 (22) | 17 (53) | 0 (0) | 0 (0) |
|  | Colloid solution | 26 (33) | 3 (9) | 0 (0) | 23 (62) |
|  | Combined | 14 (18) | 7 (22) | 1 (10) | 6 (16) |
|  | Different groups | 2 (3) | 1 (3) | 0 (0) | 1 (3) |
|  | Blood | 9 (11) | 0 (0) | 9 (90) | 0 (0) |
|  | Not reported | 11 (14) | 4 (13) | 0 (0) | 7 (19) |
|  |  |  |  |  |  |
| Priming Volume [ml] -  median (quartiles) | | 12 (10, 16) | 14 (12, 20) | 13 (13, 13) | 10 (10, 13) |
|  |  |  |  |  |  |
| Blood transfusion - n (%) | |  |  |  |  |
|  | Yes | 19 (24) | 6 (19) | 10 (100) | 3 (8) |
|  | No/not reported | 60 (76) | 26 (81) | 0 (0) | 34 (92) |
|  |  |  |  |  |  |
| Active cooling - n (%) | |  |  |  |  |
|  | Yes | 50 (63) | 4 (13) | 9 (90) | 37 (100) |
|  | Flush after cardiac arrest | 9 (18) | 0 (0) | 9 (100) | 0 (0) |
|  | Continuous cooling | 41 (82) | 4 (100) | 0 (0) | 37 (100) |
|  | No/not reported | 29 (37) | 28 (88) | 1 (10) | 0 (0) |
|  |  |  |  |  |  |
| Maximum extracorporeal target flow [ml/kg/min/] - n (%) | | |  |  |  |
|  | <50 | 2 (3) | 2 (6) | 0 (0) | 0 (0) |
|  | 50-90 | 6 (8) | 5 (16) | 0 (0) | 1 (3) |
|  | 90-130 | 19 (24) | 9 (28) | 1 (10) | 9 (24) |
|  | 130-170 | 13 (16) | 10 (31) | 0 (0) | 3 (8) |
|  | 170-190 | 19 (24) | 0 (0) | 4 (40) | 15 (41) |
|  | Not reported | 20 (25) | 6 (19) | 5 (50) | 9 (24) |
|  |  |  |  |  |  |
| Drugs used during ECLS - n (%) | |  |  |  |  |
|  | Sodium bicarbonate | 31 (39) | 15 (47) | 3 (30) | 13 (35) |
|  | Norepinephrine | 13 (16) | 5 (16) | 0 (0) | 8 (22) |
|  | Trometamol | 9 (11) | 4 (13) | 0 (0) | 5 (14) |
|  | Epinephrine | 8 (10) | 8 (25) | 0 (0) | 0 (0) |
|  | Magnesium | 4 (5) | 4 (13) | 0 (0) | 0 (0) |
|  | Calcium | 4 (5) | 0 (0) | 2 (20) | 2 (5) |
|  | Potassium | 1 (1) | 0 (0) | 1 (10) | 0 (0) |
|  | Furosemide | 1 (1) | 0 (0) | 0 (0) | 1 (3) |
|  | Dopamine | 1 (1) | 0 (0) | 0 (0) | 1 (3) |
|  |  |  |  |  |  |
|  |  |  |  |  |  |
| **Endpoint - n (%)** | |  |  |  |  |
|  | Survival | 41 (52) | 10 (31) | 9 (90) | 22 (59) |
|  |  |  |  |  |  |
|  | Neurological outcome | 50 (63) | 16 (50) | 10 (100) | 24 (65) |
|  | Cardiological outcome | 28 (35) | 16 (50) | 3 (30) | 9 (24) |
|  | Renal outcome | 4 (5) | 2 (6) | 0 (0) | 2 (5) |
|  | Other | 18 (23) | 12 (38) | 0 (0) | 6 (16) |
|  |  |  |  |  |  |
|  | ** All models performed hemorrhagia before induction of ischemia; ** see supplement for details; n, number; min, minutes; ml, milliliters; ECLS, extracorporeal life support; ECPR, Extracorporeal cardiopulmonary resuscitation; EPR, Emergency preservation and resuscitation; DHCA, Deep hypothermic circulatory arrest;* | | | | |

**Supplement 9: Recommended core dataset for reporting rat ECLS studies after global ischemia.** Essential data are indicated in boldface type; desirable data are indicated in italic type.

| **Domain** | **Item** | **What to report** | **Applies to** |
| --- | --- | --- | --- |
| **Animal characteristics** | **Species** | Species; strain/substrain; source/vendor; health status; genetic background if modified. | All |
|  | **Sex** | Sex of each animal; justification if single-sex. | All |
|  | **Weight** | Body weight at baseline (range); inclusion/exclusion thresholds if used. | All |
|  | **Morbidity** | Healthy vs disease model; induction method and duration of morbidity; genotype/phenotype if applicable. | All |
|  | *Fasting/alimental status* | Fasted vs non-fasted; fasting duration; access to water. | All |
|  | *Age* | Age in weeks (range) or exact age. | All |
| **Study design** | **Group structure** | Total animals; number of groups; animals per group (planned and analysed); definition of SHAM/control. | All |
|  | **Randomization** | Whether randomization was used; method of randomization (e.g. computer-generated, block randomization); allocation if applicable. | All |
|  | **Blinding** | Blinding of operators/outcome assessors (surgery, care, assessment, analysis). | All |
|  | *Sample size* | Sample size rationale/calculation; inclusion/exclusion criteria. | All |
|  | *Experimental timeline* | Timeline diagram or text including baseline, ischemia, ECLS, interventions, follow-up, euthanasia. | All |
| **Anaesthesia & perioperative care** | **Anaesthesia regimen** | Agent; dose/concentration; route; timing/sequence; maintenance method; ventilation settings. | All |
|  | **Analgesia regimen** | Pre-, intra- and post-procedural analgesia; agent, dose/concentration, route, interval; duration. | All |
|  | **Neuromuscular blockade (NMBA)** | Agent; dose/concentration; indication; monitoring; explicit confirmation of adequate anaesthesia/analgesia when NMBA used. | All |
|  | **Physiological monitoring** | Variables monitored and devices used: MAP (site), ECG, temperature (site), blood gases (sampling site), SpO2/EtCO2 if available. | All |
|  | **Temperature control** | Warming/cooling methods outside ECLS; target ranges; prevention of unintended hypothermia. | All |
|  | *Depth of anaesthesia* | Methods used for assessment (reflexes, physiologic criteria); frequency; actions taken if inadequate. | All |
|  | *Stabilization period* | Stabilization period before baseline measurements; predefined criteria for physiological stability. | All |
| **Ischemia induction** | **Definition of ischemia/cardiac arrest** | Operational definition (e.g., MAP threshold and duration/ECG criteria); method of validation (e.g. MAP/ECG criteria). | All |
|  | **Induction method** | Asphyxia/electrical VF/cardioplegia/hypothermia/other; protocol details; time stamps. | All |
|  | **No-flow time** | Report total no-flow duration. | All |
|  | *Ventilation settings* | Ventilator settings prior to ischemia; ventilation or apnoea strategy during ischemia; FiO2; PEEP; respiratory rate; tidal volume. | All |
| **Extracorporeal circulation**  **setup** | **Equipment identification** | manufacturer of equipment (name, model number, city, country); Tubing (material, ID, length); reservoir; pump type/model; oxygenator model. | All |
|  | **Cannulation sites** | Drainage and return sites; cannula size/length/fenestration; exact cannula placement; anticoagulation for cannulation. | All |
|  | **Oxygenator characteristics** | Gas exchange surface area; priming volume; presence of heat exchanger; priming volume; fiber type; resistance/pressure limits if known; validation data if custom made oxygenator used. | All |
|  | **Priming strategy** | Priming type (crystalloid/colloid/blood/combined); composition; total volume; temperature; blood donor origin if used. | All |
|  | **Anticoagulation** | Agent; dose/concentration; route; timing; targets (ACT/aPTT); reversal if any. | All |
|  | **Gas management** | Sweep gas flow; oxygen fraction (FiO2); CO2 management; sampling strategy to adjust settings; predefined target ranges for O_2_/CO_2_ partial pressure. | All |
|  | **Flow targets and measurement** | Target flow (ml/kg/min, ml/min); Pump speed; flow measurement method and time points, actions taken if inadequate. | All |
|  | **Temperature management** | Cooling/rewarming device used; adjustment measures; gradients; time points. | All |
|  | *Circuit pressures* | Arterial/venous line pressures; alarms/safety measures; haemolysis mitigation. | All |
| **Management during ECLS** | **Vasoactive and metabolic drugs** | Agent; dose/concentration; route; timing; trigger and predefined target (e.g. MAP, pH). | All |
|  | **Volume and transfusion management** | Fluids/blood products/Priming; volumes; product type; autologous vs donor blood; trigger and predefined target (e.g. Hb, MAP). | All |
|  | **Ventilation during ECLS** | Ventilator settings or apnoea strategy; FiO2; PEEP; respiratory rate; tidal volume. | All |
|  | **Duration on ECLS** | Total time on extracorporeal life support per animal (min); report planned versus actual duration if deviating. | All |
|  | *Additional drugs* | Indication/trigger; agent; dose; concentration; route; timing/sequence. | All |
| **Model characteristics** | **CPR/ALS procedures** | Chest compression method and rate/depth/compression waveform/compression point/physiologic targets; total low flow time; ventilation during CPR; defibrillation strategy (energy and number); drugs (dose/concentration, route, timing); criteria to initiate ECLS; chest compression quality parameters (e.g. etCO_2_ , MAP, CPP). | ECPR |
|  | **ROSC** | Define ROSC (criteria and duration) when applicable; | ECPR/EPR |
|  | **Haemorrhage protocol** | Blood withdrawal volume (mL and % estimated blood volume); withdrawal rate and duration; duration of haemorrhagic shock before cardiac arrest; target MAP; duration of “flush” cardiac arrest; additional trauma/injury (if applicable). | EPR |
|  | **Flush cooling protocol** | Flush solution; volume; temperature; route incl. draining route; timing relative to arrest and ECLS start. | EPR |
|  | **Cooling/rewarming profile** | Target temperature for each experimental phase; site of measurement; cooling and rewarming rates; cooling and rewarming techniques; duration of each period. | All |
|  | **Flow targets** | Target flow for different experimental phases (ml/kg/min, ml/min) and actions taken if inadequate; Pump speed; duration of different experimental phases. | DHCA/EPR |
|  | *Weaning/termination criteria* | Criteria to stop ECLS; transition strategy; post-ECLS support; euthanasia criteria if non-recoverable. | All |
|  | *Residual perfusion* | Report whether residual native cardiac perfusion/native cardiac output is maintained alongside extracorporeal circulation; specify when (experimental phase/time points), extent (if quantified), and assessment method (e.g. arterial pulsatility/echocardiography/flow measurements). | DHCA |
|  | *Adverse events/complications* | Bleeding, thrombosis, haemolysis, limb ischemia, circuit failure; how handled; exclusions. | All |
| **Data acquisition and reporting** | **Arterial pressure** | Report MAP/SAP/DAP; measurement site; sampling frequency; for baseline and key time points. | All |
|  | **Blood gas analysis** | Sampling site; time points; pH, PaO2/PaCO2, lactate, electrolytes; Hb/haematocrit; for baseline and key time points. | All |
|  | **Temperature** | Measurement site; continuous vs intermittent; baseline data prior to ischemia, ECLS, cooling and rewarming time points. | All |
| **Transparency** | **Reporting checklists and data access** | ARRIVE 2.0 compliance statement; protocol registration (if any); data/code availability. | All |

*ECLS, extracorporeal life support; ECPR, extracorporeal cardiopulmonary resuscitation; EPR, emergency preservation and resuscitation; DHCA, deep hypothermic circulatory arrest; ROSC, return of spontaneous circulation; MAP, mean arterial pressure; DAP, diastolic arterial pressure; SAP, systolic arterial pressure; etCO2, end-tidal carbon dioxide; CPP, coronary perfusion pressure; ACT, activated clotting time; NMBA, neuromuscular blocking agent; CPR, cardiopulmonary resuscitation; ALS, advanced life support; ml, millilitres; kg, kilogram; min, minutes; PEEP, positive end-expiratory pressure; ECG, electrocardiogram.*
